# Bioadhesive hydrogels as immunomodulatory interfaces for chronic wound healing: from microenvironmental regulation mechanism to engineering strategies

**DOI:** 10.1093/burnst/tkag055

**Published:** 2026-08-22

**Authors:** Kayoung Son, Soo A Kim, Minkyong Kang, Yejin Jo, Chansoo Kim, Kijun Park, Yunmin Jung, Tae Young Kim, Jungmok Seo

**Affiliations:** School of Electrical and Electronic Engineering, Yonsei University, 50 Yonsei-ro, Seodaemun-gu, Seoul 03722, Republic of Korea; School of Electrical and Electronic Engineering, Yonsei University, 50 Yonsei-ro, Seodaemun-gu, Seoul 03722, Republic of Korea; School of Electrical and Electronic Engineering, Yonsei University, 50 Yonsei-ro, Seodaemun-gu, Seoul 03722, Republic of Korea; School of Electrical and Electronic Engineering, Yonsei University, 50 Yonsei-ro, Seodaemun-gu, Seoul 03722, Republic of Korea; School of Electrical and Electronic Engineering, Yonsei University, 50 Yonsei-ro, Seodaemun-gu, Seoul 03722, Republic of Korea; School of Electrical and Electronic Engineering, Yonsei University, 50 Yonsei-ro, Seodaemun-gu, Seoul 03722, Republic of Korea; Division of Engineering in Medicine, Department of Medicine, Brigham and Women’s Hospital, Harvard Medical School, 65 Landsdowne Street, Cambridge, MA 02139, United States; School of Electrical and Electronic Engineering, Yonsei University, 50 Yonsei-ro, Seodaemun-gu, Seoul 03722, Republic of Korea; School of Electrical and Electronic Engineering, Yonsei University, 50 Yonsei-ro, Seodaemun-gu, Seoul 03722, Republic of Korea; School of Electrical and Electronic Engineering, Yonsei University, 50 Yonsei-ro, Seodaemun-gu, Seoul 03722, Republic of Korea

**Keywords:** Chronic wounds, Macrophage regulation, Bioadhesive hydrogel, Immunomodulatory hydrogels, Multifunctional hydrogels, Bioactive material

## Abstract

Chronic wounds represent a major and growing clinical burden, particularly among aging populations and individuals with diabetes, in whom persistent inflammation and immune dysregulation delay normal tissue repair. Addressing this challenge requires functional materials that do more than passively cover the wound and instead actively regulate the wound microenvironment. Advances in hydrogel chemistry and bioengineering have enabled the development of bioadhesive interfaces that maintain stable contact with wound tissue while modulating immune and regenerative responses. In this Review, we present bioadhesive hydrogels as immunomodulatory interfaces rather than simple wound coverings and systematically examine their mechanisms of action, including the reduction of microbial and inflammatory stimuli; modulation of macrophage polarization; regulation of NF-κB-, NLRP3-, and STAT-associated signaling; and control of mechanobiological signaling at the material–tissue interface. Through these mechanisms, bioadhesive hydrogels can alleviate excessive inflammation and support a regenerative microenvironment that promotes angiogenesis, re-epithelialization, and extracellular matrix remodeling. We classify the engineering approaches used to achieve these functions into four complementary strategies. Structural and protective hydrogels stabilize the wound and protect it from contamination and mechanical disruption. Bioactive and regenerative systems deliver therapeutic molecules or cells to regulate inflammation and promote repair. Externally activated platforms provide spatiotemporal control over therapeutic activity, whereas sensing-integrated systems monitor wound conditions and support adaptive or closed-loop treatment. We also discuss major translational barriers, including unstable adhesion under exudative and mechanically dynamic conditions, limited long-term safety data, insufficient standardization, manufacturing challenges, and unclear regulatory pathways. Future progress will require disease-specific material design, clinically relevant validation, and the reliable integration of sensing and therapeutic functions.

## Graphical Abstract

**Graphical Abstract:**
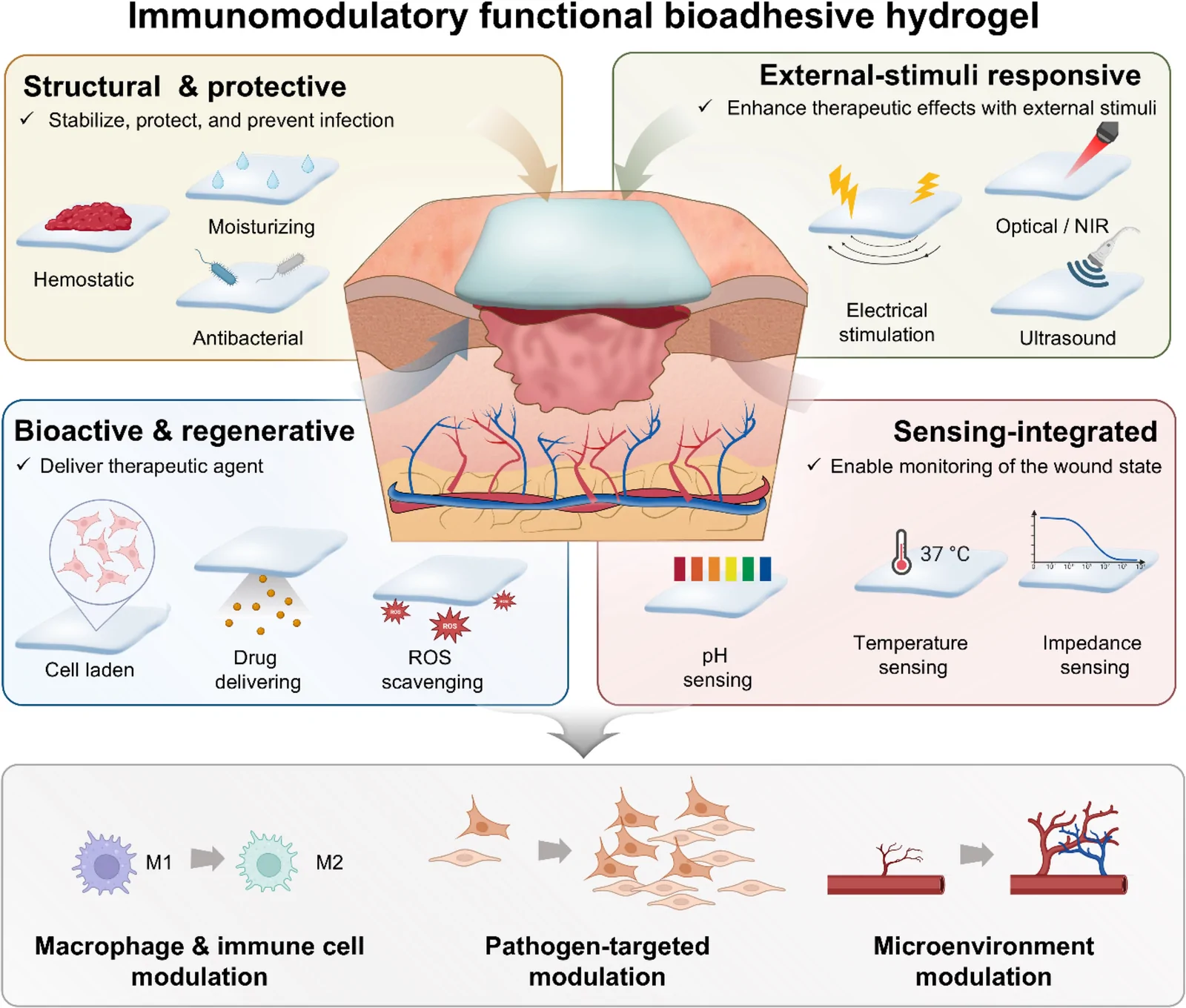

## Highlights

- Reframes hydrogel bioadhesives as immunomodulatory interfaces for chronic wounds.
- Addresses the failure of coordinated hemostasis, inflammation resolution, proliferation, and remodeling that underlie chronic wound pathology.
- Defines key property requirements of hydrogels for wet, exudative, and dynamic wound settings.
- Organizes recent advances into four functional dimensions: structural/protective design, bioactive and regenerative strategies, externally triggered platforms, and sensing-integrated systems.
- Addresses translational challenges in stability, safety, manufacturing, and regulation.

## Background

Chronic wounds impose a growing and substantial clinical burden, particularly among aging populations and patients with metabolic disorders such as diabetes [1, 2]. Unlike acute wounds, which progress through tightly regulated phases of hemostasis, inflammation, proliferation, and remodeling, chronic wounds fail to resolve inflammation promptly and become trapped in a pathological inflammatory state [3]. This persistent immune dysregulation delays tissue regeneration, which accelerates extracellular matrix (ECM) degradation, compromises barrier integrity, and ultimately leads to nonhealing lesions that are highly susceptible to infection and recurrence [4].

A key contributor to this impaired healing trajectory is the breakdown of immune resolution mechanisms, particularly the dysregulation of macrophage phenotypic transitions. During physiological wound repair, macrophages undergo a coordinated shift from a pro-inflammatory M1 phenotype to a pro-regenerative M2 phenotype as healing progresses [5, 6]. This phenotypic plasticity is essential for early pathogen and debris clearance, followed by promotion of angiogenesis, fibroblast activation, and matrix deposition in later stages. In chronic wounds, however, this transition is markedly impaired [7]. Persistent M1 polarization drives the sustained secretion of pro-inflammatory cytokines, matrix metalloproteinases, and reactive oxygen species (ROS), thereby exacerbating tissue damage, suppressing the function of fibroblasts and endothelial cells, and hindering the establishment of a regenerative microenvironment [8–10]. These immune abnormalities are further sustained by excessive exudate, bacterial colonization and biofilm formation, hypoxia, abnormal pH, protease imbalance, and repeated mechanical disruption, creating a self-perpetuating cycle of inflammation and tissue damage [11].

Conventional wound dressings can provide essential physical protection and moisture management, but most remain predominantly passive and offer limited control over the persistent inflammatory, microbial, and biochemical abnormalities of chronic wounds. Their insufficient conformity and retention on wet, irregular, and mechanically active wound surfaces may also create interfacial gaps or premature detachment, reducing continuous protection and local therapeutic efficacy [12]. Effective chronic wound management, therefore, requires materials that can establish stable contact with the wound while actively regulating this complex pathological environment [13]. In this context, bioadhesion should be viewed not merely as a strategy for retaining a dressing on the tissue but as a means of creating a continuous functional interface between the material and the wound bed. Stable and conformal adhesion can reduce interfacial gaps, fluid leakage, microbial entry, and micromotion-induced damage while maintaining localized transport of therapeutic molecules and sustained interactions with immune and regenerative cells [14].

Hydrogel-based bioadhesives have emerged as particularly promising platforms for engineering such immunoactive wound interfaces. Owing to their high water content, tissue-like softness, and tunable chemical functionality, hydrogels closely mimic the native ECM while maintaining conformal contact with wet biological tissues [15, 16]. Their hydrated polymer networks facilitate nutrient diffusion and cytokine transport, while mechanical compliance minimizes stress concentration at the wound edge [17, 18]. Moreover, their composition, network structure, degradation behavior, and interfacial properties can be tailored to regulate not only wound coverage and moisture balance but also oxidative stress, microbial activity, cellular signaling, and tissue regeneration.

**Figure 1:**
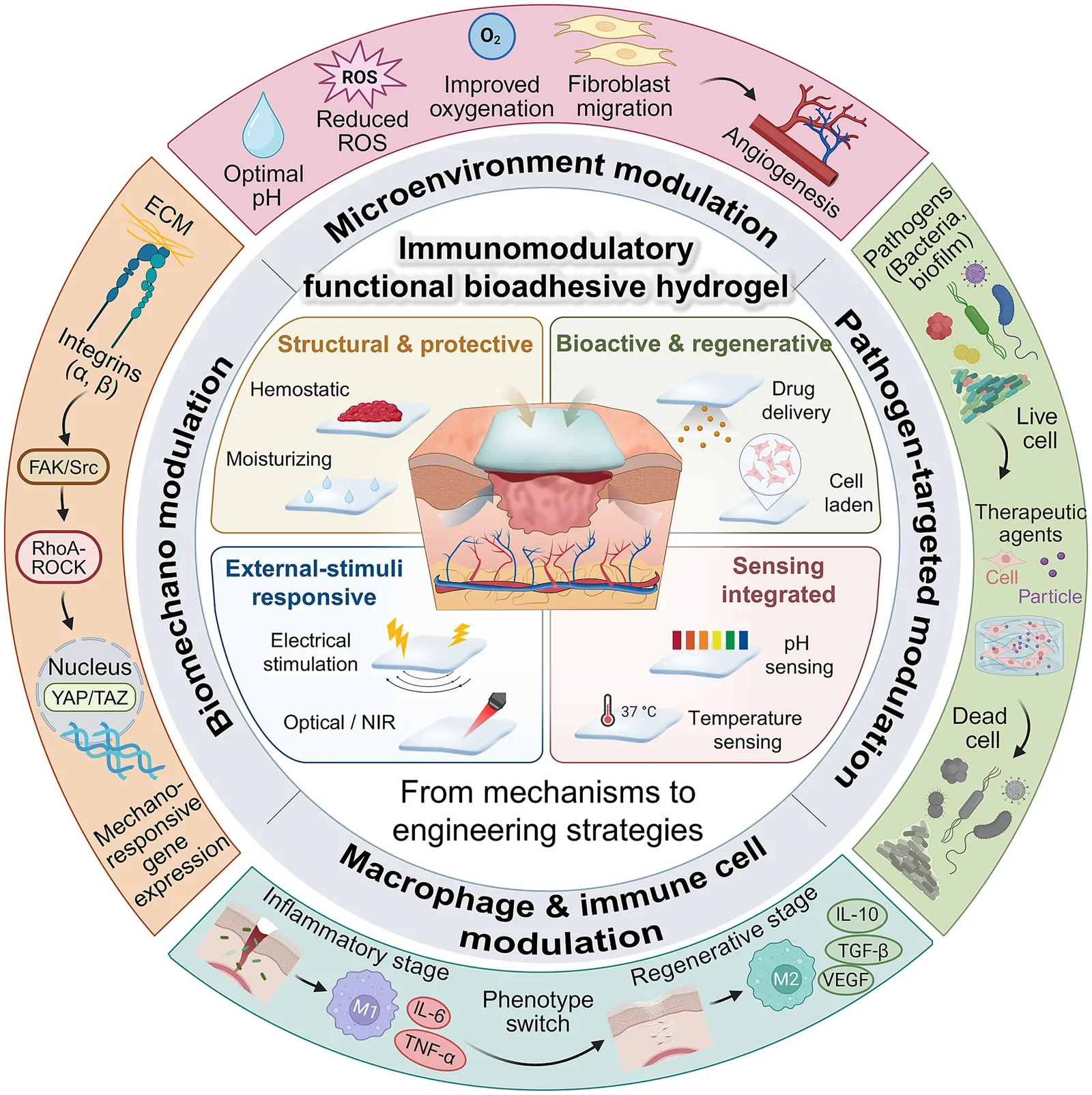
Immunomodulatory functional bioadhesive hydrogels for advanced wound healing. Schematic overview of bioadhesive hydrogel interfaces designed to regulate the chronic wound microenvironment through interconnected mechanisms, including microenvironment modulation, pathogen control, immune-cell regulation, and mechanobiological signaling. *ECM* extracellular matrix, *TNF* tumor necrosis factor alpha, *IL* interleukin, *VEGF* vascular endothelial growth factor, *NIR* near-infrared, *ROS* reactive oxygen species

As illustrated in Figure 1, bioadhesive hydrogels can influence wound healing through several interconnected mechanisms, including modulation of the biochemical microenvironment, reduction of pathogen-associated inflammatory stimuli, regulation of macrophage and broader immune-cell responses, and mechanobiological signaling at the material–tissue interface. These effects may restore more favorable levels of ROS, oxygenation, and pH; support fibroblast migration and angiogenesis; promote the transition from persistent inflammatory activity toward reparative immune states; and regulate cell behavior through ECM–integrin-associated mechanotransduction. Rather than functioning independently, these mechanisms collectively reshape the chronic wound microenvironment and facilitate progression toward tissue repair.

Recent advances have transformed bioadhesive hydrogels from simple wound sealants into multifunctional therapeutic interfaces [19]. In this review, we connect these biological mechanisms with four complementary engineering strategies: structural and protective bioadhesives that stabilize and shield the wound; bioactive and regenerative systems that deliver therapeutic agents or living components; externally activated platforms that provide spatial and temporal control over treatment; and sensing-integrated systems that enable wound monitoring and adaptive intervention. By organizing diverse hydrogel technologies according to both their immunomodulatory mechanisms and their engineering functions, this framework highlights how bioadhesion supports the coordinated regulation of the wound microenvironment rather than serving only as a fixation mechanism.

We first describe the pathological and immunological features of chronic wounds and the functional requirements of bioadhesive hydrogel interfaces. We then examine the four engineering strategies outlined above, with particular attention to their effects on inflammatory resolution, pathogen control, mechanobiological signaling, and tissue regeneration. Representative conventional dressings and advanced bioadhesive platforms are further comparatively summarized in Tables according to their key material characteristics, therapeutic performance, experimental models, and translational considerations. Finally, we discuss the major translational challenges associated with long-term interfacial stability, biological safety, manufacturing scalability, standardization, and regulatory approval, and consider future directions toward adaptive and closed-loop wound-care systems.

## Review

### Hydrogel-based bioadhesive for immunomodulation

#### Phase-specific immune regulation and its dysregulation in chronic wounds

**Figure 2:**
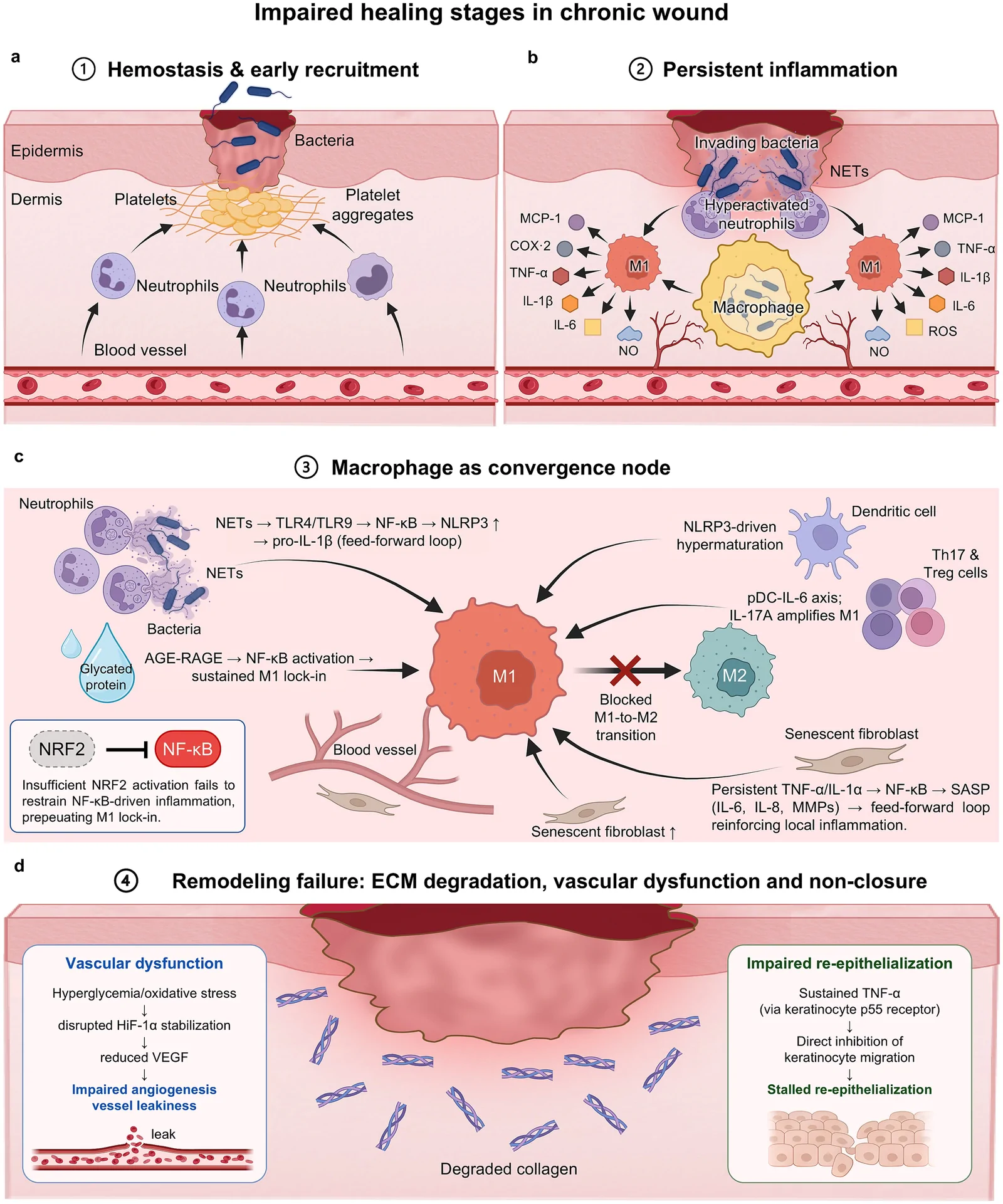
Phase-specific immune dysregulation and impaired healing mechanisms in chronic wounds. (**a**) Hemostasis phase showing platelet aggregation, temporary wound coverage, and initial immune cell recruitment. (**b**) Persistent inflammatory phase showing bacterial biofilm colonization, NET formation, and M1 macrophage activation leading to the continuous release of diverse pro-inflammatory mediators. (**c**) Macrophages integrate dysregulated upstream signals from NETs, glycated proteins, T-cell subsets, dendritic cells, and senescent fibroblasts (SASP). Insufficient NRF2 restraint perpetuates NF-κB activation, blocking the M1-to-M2 transition. (**d**) Impaired tissue remodeling characterized by vascular dysfunction via disrupted HIF-1α and VEGF signaling, alongside stalled re-epithelialization driven by sustained TNF-α signaling. *ECM* extracellular matrix, *TNF* tumor necrosis factor alpha, *IL* interleukin, *VEGF* vascular endothelial growth factor, *NIR* near-infrared, *ROS* reactive oxygen species

Cutaneous wound healing normally proceeds through the coordinated phases of hemostasis, inflammation, proliferation, and remodeling, ultimately leading to restoration of tissue integrity [20–22]. In contrast, chronic wounds fail to progress efficiently through these sequential stages and instead remain trapped in a prolonged inflammatory state. This pathological microenvironment is characterized by severe cellular dysfunction, persistent microbial colonization, and a highly proteolytic and oxidative milieu that actively degrades newly formed tissues and therapeutic biomolecules. To clarify how this dysregulation develops, the following section first outlines the key cellular and molecular events that define each phase of normal wound healing. Immediately after tissue injury, vascular disruption leads to bleeding and rapid clot formation at the wound site, where platelets and their aggregates quickly assemble to form a hemostatic plug. This plug limits blood loss, provides temporary coverage of the wound bed, and supports subsequent immune cell recruitment from the sinusoidal circulation (Figure 2a) [23, 24].

Following hemostasis, the wound bed becomes highly susceptible to pathogen invasion, which, in chronic wounds, rapidly transitions into a state of persistent, nonresolving inflammation (Figure 2b). As the first line of defense, neutrophils are recruited early to the site. However, rather than simply clearing pathogens and safely undergoing apoptosis as they do in normal healing, these cells become hyperactivated and exhibit a prolonged residence time. This sustained activation leads to the excessive generation of neutrophil extracellular traps (NETs). These web-like structures of DNA and proteins are designed to ensnare invading bacteria, but they paradoxically inflict severe secondary damage to the surrounding host tissue [25]. Concurrently, the chronic wound bed is frequently colonized by bacterial biofilms, which create a physical and biochemical shield that sustains a high local antigen load and severely frustrates immune clearance mechanisms [26]. Recruited into this hostile, pathogen-rich environment, monocyte-derived macrophages predominantly polarize into the classical pro-inflammatory (M1) phenotype. These M1 macrophages secrete a massive array of pro-inflammatory mediators, including MCP-1, COX-2, TNF-α, IL-1β, IL-6, nitric oxide (NO), and ROS. All of these act synergistically to amplify local tissue inflammation [22, 27, 28]. While this intense inflammatory burst is acute and self-limiting in normal healing, these cues persistently circulate in chronic wounds. Crucially, this persistence is not solely driven by a macrophage-intrinsic defect. Rather, a complex network of surrounding immune cells, including neutrophils, dendritic cells, and specific T-cell subsets, continuously feeds inflammatory signals back into the microenvironment [29].

Mechanistically, this multifaceted inflammatory drive converges directly on the macrophage, establishing it as a central signaling node that integrates multiple dysregulated upstream inputs (Figure 2c). First, the previously mentioned NETs shed components that continuously bind to Toll-like receptors (TLR4 and TLR9) on the macrophage surface. This binding triggers NF-κB, a master transcription factor that acts as the primary “on-switch” for cellular inflammation. The activation of NF-κB subsequently drives the assembly of the NLRP3 inflammasome, a multi-protein intracellular complex responsible for processing and releasing potent inflammatory cytokines. Together, they create a vicious feed-forward loop of pro-IL-1β production [30]. In parallel, chronic wounds are often complicated by underlying systemic conditions such as hyperglycemia, which leads to the local accumulation of advanced glycation end-products (AGEs). These glycated proteins engage their specific receptor (RAGE) on macrophages, delivering another potent stimulus for NF-κB activation that actively locks the cells in the M1 state. Under normal physiological conditions, a transcription factor known as NRF2 acts as a crucial brake to restrain this NF-κB-driven inflammation. However, in the severely compromised chronic wound microenvironment, this NRF2-mediated restraint is functionally insufficient, allowing the inflammatory lock-in to proceed unchecked [31].

Beyond these direct molecular triggers, the macrophage is also heavily influenced by paracrine signals from neighboring cells. Dendritic cells in this environment undergo an abnormal, NLRP3-driven hypermaturation, establishing a plasmacytoid dendritic cell (pDC)-IL-6 axis. When combined with excessive IL-17A secreted by an abnormally expanded Th17 cell population and a relative deficiency of inflammation-suppressing regulatory T (Treg) cells, this signaling milieu massively amplifies M1 polarization [32]. Furthermore, structural cells also participate in this detrimental loop. Senescent fibroblasts, which accumulate in chronic wounds due to cellular exhaustion, are persistently exposed to local TNF-α and IL-1α. This exposure drives NF-κB activation within the fibroblasts themselves, prompting them to adopt a senescence-associated secretory phenotype (SASP). The SASP is characterized by the continuous release of IL-6, IL-8, and matrix metalloproteinases (MMPs), serving as yet another powerful feed-forward signal that reinforces macrophage M1 polarization [25].

Collectively, this simultaneous convergence of hyperactive signals from neutrophils, dendritic cells, lymphocytes, and senescent fibroblasts onto the macrophage perfectly explains why the critical transition from the inflammatory M1 to the reparative M2 phenotype remains completely blocked in chronic wounds. It also clearly illustrates why targeted immunomodulation of macrophages remains a highly rational and potent therapeutic strategy, even given the overwhelming cellular complexity of the wound bed [33, 34]. For context, in normal wound healing, macrophages easily escape these signals and complete their phenotypic shift to the reparative M2 state, where they secrete pro-healing factors like TGF-β, IL-10, IL-1Ra, PDGF, and Vascular Endothelial Growth Factor (VEGF) to support essential downstream processes such as angiogenesis, fibroblast proliferation, and granulation tissue formation [35, 36].

The consequences of this blocked transition extend into a failure of coordinated tissue remodeling (Figure 2d). Hyperglycemia and the associated oxidative stress destabilize HIF-1α signaling. This reduces VEGF expression, impairing angiogenesis and promoting vascular leakiness [37]. At the same time, sustained TNF-α signaling through the keratinocyte p55 receptor directly inhibits keratinocyte migration, stalling re-epithelialization [38]. Excessive MMP activity further degrades disorganized ECM components. Together, these processes prevent the controlled collagen remodeling and scar maturation seen in normal wounds [39–42]. Consequently, normal wounds progress toward stable wound closure, whereas chronic wounds often remain incompletely closed and structurally unresolved [43]. Taken together, these findings indicate that chronic wounds are not simply delayed versions of normal wounds. They are pathological wounds in which the normal sequence of hemostatic stabilization, inflammatory resolution, reparative activation, and matrix remodeling is fundamentally disrupted. This disruption is driven by a multicellular, self-reinforcing inflammatory network converging on the macrophage.

Ultimately, overcoming these deeply entrenched pathological barriers requires more than passive dressings. There is an urgent clinical need for advanced biomaterials capable of actively regulating this dysregulated microenvironment, restoring macrophage polarization dynamics, and orchestrating a shift back toward a pro-healing state [44].

### Design principles of bioadhesive hydrogels for immunomodulation

#### Required properties of bioadhesive hydrogels in clinical practices

**Figure 3:**
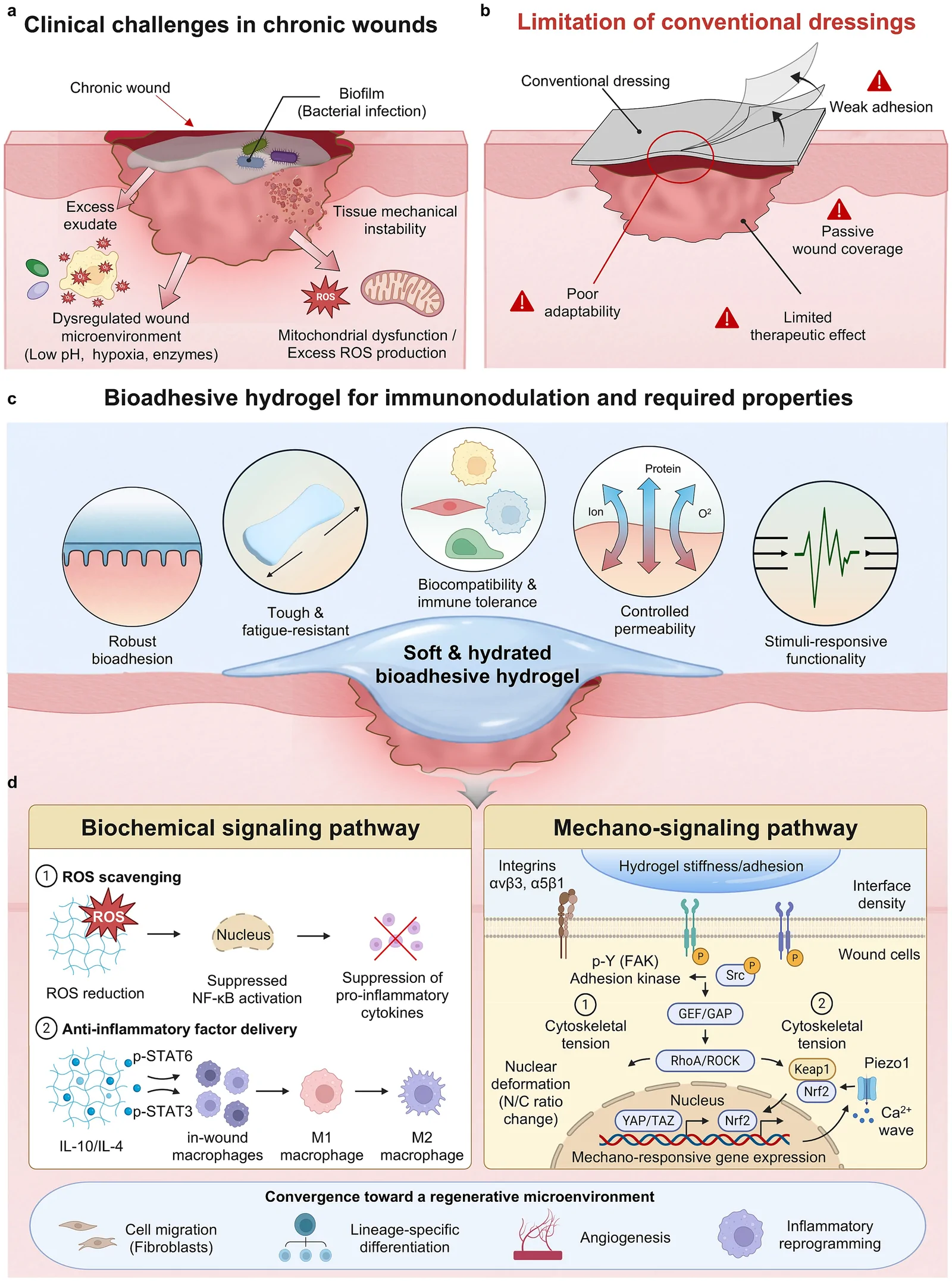
Design considerations and representative immunomodulatory mechanisms of bioadhesive hydrogels in clinically relevant chronic wound environments. (**a**) Schematic illustration of chronic wounds, characterized by excess exudate, persistent inflammatory signals, and a dysregulated wound microenvironment. (**b**) Schematic illustration of conventional wound dressings exhibits weak adhesion, poor adaptability to irregular wound geometries, passive wound coverage, and consequently limited therapeutic effect. (**c**) Required properties of immunomodulatory bioadhesive hydrogels, including robust bioadhesion, toughness and fatigue resistance, biocompatibility and immune tolerance, controlled permeability, stimuli-responsive functionality, and a soft, hydrated structure. (**d**) Representative biochemical and mechano-signaling pathways associated with bioadhesive hydrogel-mediated immunomodulation. ROS scavenging, anti-inflammatory factor delivery, and mechanotransduction-related signaling collectively promote macrophage reprogramming, cell migration, differentiation, and angiogenesis. The level of experimental validation varies among individual hydrogel systems

As outlined in the previous section, chronic wounds are clinically characterized by a persistently dysregulated wound environment that fails to progress through the normal sequence of healing. In particular, they are associated with excess exudate, persistent inflammatory signals, and a dysregulated wound microenvironment characterized by abnormal pH, hypoxia, and excessive enzymatic activity (Figure 3a). Excess exudate destabilizes the local wound environment, compromises dressing adhesion, and increases the risk of tissue maceration and infection [45]. At the same time, inflammatory mediators such as cytokines, ROS, and proteolytic enzymes accumulate, sustaining persistent inflammatory signals that prolong the M1-dominant macrophage state and hinder the transition toward tissue repair. This pathological milieu further disrupts essential cellular functions, including proliferation, migration, and matrix remodeling, ultimately preventing proper healing progression [42].

Conventional wound dressings have primarily provided physical protection, moisture regulation, exudate absorption, cushioning, and autolytic debridement, depending on the dressing type. Gauze dressings are inexpensive, widely available, and easy to apply, but they provide limited moisture regulation and adhere to newly formed tissue, causing pain or secondary injury during removal [46]. Transparent films enable visual inspection and provide a water-resistant barrier, but their minimal absorption capacity restricts their use to superficial wounds with low exudate levels [47]. Foam dressings offer effective exudate absorption, cushioning, and thermal insulation, whereas hydrocolloid dressings maintain a moist environment and support autolytic debridement. Nevertheless, these dressings exhibit insufficient conformability or fluid-handling capacity in highly irregular, infected, or heavily exudative wounds [48]. Alginate dressings provide high absorption capacity and can support hemostasis in bleeding wounds, but they generally require secondary fixation and may desiccate wounds with limited exudate [49]. Conventional non-adhesive hydrogel dressings provide hydration, cooling, and pain relief and can soften dry necrotic tissue, although their relatively weak mechanical integrity, limited exudate-handling capacity, and poor tissue retention restrict prolonged application [46].

Although these dressing categories offer distinct clinical advantages according to wound depth, exudate level, infection status, and treatment stage, their functional limitations remain evident. Most conventional dressings are optimized for one or a limited number of passive functions, such as wound coverage, fluid management, cushioning, or moisture maintenance, rather than the coordinated regulation of complex chronic wound environments [11]. Many exhibit insufficient adhesion to wet and irregular wound surfaces and undergo edge lifting, interfacial gap formation, or premature detachment under dynamic conditions (Figure 3b) [50]. Poor conformability reduces continuous contact with the wound bed, compromises barrier integrity, and limits the local retention of endogenous or delivered therapeutic factors. Moreover, conventional dressings generally provide limited control over inflammatory mediators, oxidative stress, microbial burden, and molecular transport. These clear limitations have motivated the development of bioadhesive hydrogels capable of combining stable tissue attachment with active and sustained regulation of the wound microenvironment [44–52] (Table 1).

**Table 1:** Classification of conventional wound dressing types and their advantages, limitations, and functional gap relative to bioadhesive immunomodulatory hydrogels

Dressing type	Typical materials/ form	Major clinical advantages	Principal limitations	Suitable wound conditions	Limitations relative to bioadhesive hydrogels	Refs.
Gauze dressings	Woven or nonwoven cotton/rayon	Low cost, widely available, easy application	Requires frequent replacement, limited moisture control, and barrier function	Superficial wounds or secondary dressings	Poor conformability and wet adhesion; no active microenvironment or immune regulation	[46]
Transparent films	Polyurethane films	Flexible, transparent, water-resistant, and permits visual inspection	Minimal exudate absorption; risk of fluid accumulation and maceration	Superficial, low-exudate wounds	Limited use in irregular or highly exudative chronic wounds	[47]
Foam dressings	Polyurethane or silicone foam	High exudate absorption, cushioning, thermal insulation	Dry low-exudate wounds; limited contact with deep or irregular cavities	Moderate-to-high-exudate wounds	Limited active biological function and variable fixation under motion	[48]
Hydrocolloid dressings	Gelatin, pectin, carboxymethylcellulose	Maintains a moist environment, supports autolytic debridement, relatively long wear time	Occlusive; retains excessive fluid and is less suitable for heavily infected wounds.	Low-to-moderate-exudate wounds	Limited real-time monitoring, immune modulation, and programmable therapy	[48]
Alginate dressings	Calcium or sodium alginate fibers	High absorbency, conformability, and potential hemostatic function	Requires secondary fixation; desiccate wounds with limited exudate	Bleeding or highly exudative wounds	Weak intrinsic tissue adhesion and limited structural stability	[49]
Conventional hydrogel dressings	Water-rich natural or synthetic polymer networks	Cooling, hydration, pain relief, and softening of necrotic tissue	Low mechanical strength, limited exudate capacity, weak tissue retention	Dry, painful, or necrotic wounds	Generally lacks durable wet adhesion, active immunomodulation, and adaptive sensing.	[46].
Bioadhesive immunomodulatory hydrogels	Adhesive and multifunctional polymer networks	Conformal wet adhesion, localized delivery, immune regulation, optional sensing, and stimuli responsiveness	Greater formulation and manufacturing complexity, regulatory and cost barriers	Complex chronic wounds requiring sustained interface control	Emerging systems require stronger long-term and clinical validation	[44–52]

These advances shift the design focus from simply adding individual functions to establishing a coordinated set of material requirements that can maintain a stable wound interface while actively supporting the healing process. To function effectively in clinically relevant wound environments, bioadhesive hydrogels should integrate several complementary interfacial, mechanical, transport, and biological properties (Figure 3c). Robust wet adhesion is required to establish rapid and sustained attachment to moist tissue, whereas conformal adaptability allows the material to accommodate irregular wound geometries, fill interfacial voids, and preserve contact during tissue deformation [53]. Stable interfacial bonding should be supported by sufficient cohesive strength, toughness, and fatigue resistance to withstand repeated mechanical loading, friction, swelling, and handling during prolonged application [54, 55]. At the same time, the hydrogel should retain a soft and hydrated structure that minimizes mechanical mismatch with native tissue, reduces stress concentration at the wound margin, and maintains intimate contact without undergoing excessive deformation or washout.

Biocompatibility and immune tolerance are also essential. The polymer network, crosslinking chemistry, incorporated therapeutic components, and degradation products should not induce substantial cytotoxicity or excessive inflammatory activation [56, 57]. Ideally, the hydrogel should provide a tissue-compatible microenvironment that supports the viability and function of immune, stromal, epithelial, and vascular cells while permitting progressive tissue integration and remodeling. Appropriate degradation kinetics are also important because premature degradation compromises wound coverage, whereas prolonged persistence may interfere with tissue reconstruction or dressing removal.

Controlled permeability and moisture management are equally important for maintaining a balanced wound environment. The hydrogel network should regulate the transport of water, oxygen, ions, nutrients, proteins, cytokines, and therapeutic cargos while effectively managing exudate. Excessive swelling can increase interfacial stress, weaken cohesive integrity, and promote delamination, whereas dehydration can reduce conformability and create interfacial gaps that increase susceptibility to contamination [58]. Water uptake, retention, and release should therefore be coordinated with the mechanical stability and adhesive performance of the material. In drug- or factor-releasing systems, the network should additionally protect therapeutic cargos from premature degradation and provide release profiles compatible with the intended treatment period. Because diffusion and matrix degradation are influenced by local pH, ROS levels, enzymatic activity, and ionic conditions, release behavior should be designed in consideration of the dynamic wound microenvironment [59, 60].

Stimuli-responsive functionality can further expand the therapeutic capability of bioadhesive hydrogels. Endogenous wound-associated signals, including pH, temperature, ROS, enzymes, glucose, and inflammatory mediators, may be used to regulate network degradation, therapeutic release, antimicrobial activity, or mechanical behavior. External inputs such as light, electrical stimulation, heat, and ultrasound can also provide spatially and temporally controlled activation. These responsive properties enable the hydrogel to adjust its function according to changes in wound status and offer more precise control over the local healing environment.

However, the effective implementation of these functions requires selective customization rather than the indiscriminate integration of all available properties. Hydrogel design should therefore be tailored not only to the underlying wound subtype but also to the changing biological and mechanical demands encountered during different stages of healing.

Although stable wet adhesion, mechanical compatibility, immune tolerance, and controlled mass transport are broadly relevant, their relative importance and functional implementation should be tailored to wound etiology and clinical context. Diabetic foot ulcers may place greater emphasis on immunomodulatory, antimicrobial, pro-angiogenic, and sensing functions [61], whereas venous leg ulcers require effective exudate management and sustained adhesion under fluid-rich conditions [62]. Pressure injuries demand load redistribution and resistance to repeated compression and shear [63], while ischemic or arterial wounds require greater emphasis on hypoxia mitigation, perfusion restoration, and pro-angiogenic support [64]. Thus, subtype-specific pathophysiology and established clinical management should guide material selection, functional prioritization, and validation.

Beyond these subtype-specific requirements, hydrogel functions should also be dynamically matched to the evolving needs of the wound-healing process. Immediately after injury, bleeding, exudate accumulation, microbial exposure, and repeated tissue motion create a highly unstable interface. Rapid sealing, hemostasis, fluid management, and protection against contamination are therefore required to stabilize the wound bed. Establishing a continuous interface at this stage not only provides physical coverage but also limits the loss of blood, endogenous signaling factors, and therapeutic agents while reducing additional tissue damage caused by friction and micromotion [20].

As inflammation begins to resolve, regulation of excessive ROS, inflammatory cytokines, proteolytic enzymes, and microbial stimuli becomes increasingly important. Controlled hydration and molecular transport can limit the accumulation of damaging inflammatory mediators, preserve local concentrations of therapeutic factors, and promote a shift toward a pro-healing immune environment [22]. In chronic wounds, effective immunomodulation therefore requires not only attenuation of persistent inflammation but also restoration of coordinated interactions between immune cells and tissue-resident regenerative cells. Importantly, anti-inflammatory and antioxidant functions should be temporally regulated rather than continuously maximized. Transient inflammatory and ROS signals remain necessary for microbial clearance, immune-cell recruitment, and the initiation of regenerative signaling, whereas excessive or prolonged suppression may compromise host defense and delay subsequent repair. Hydrogel systems should therefore attenuate pathological inflammation while preserving the physiological immune and redox signals required for healing progression.

With progression into the proliferative phase, keratinocyte and fibroblast migration, endothelial activation, angiogenesis, granulation tissue formation, and ECM deposition become dominant. The hydrogel should accordingly provide suitable porosity, permeability, and degradation behavior to support cellular infiltration and migration while maintaining oxygen and nutrient transport and preserving regenerative signals [65]. As newly formed tissue gradually fills the defect, the mechanical properties, adhesive strength, and degradation rate of the hydrogel should remain compatible with the evolving tissue environment.

At the remodeling stage, the newly deposited ECM undergoes reorganization and maturation, accompanied by progressive restoration of tissue mechanical integrity. Controlled degradation and gradual transfer of mechanical load from the material to the regenerating tissue are therefore important to avoid interfering with collagen organization and functional recovery. Accordingly, clinically effective bioadhesive hydrogels should selectively integrate interfacial, protective, immunomodulatory, and regenerative functions according to wound-specific needs while remaining aligned with the evolving requirements of the healing process. The compositional and structural tunability of hydrogel networks facilitates both subtype-specific and stage-responsive control, enabling their adhesive, mechanical, transport, and therapeutic properties to be tailored to different wound etiologies and phases of healing.

#### Bioadhesive hydrogel designs for immunomodulation

The properties described in the Required Properties of Bioadh-  esive Hydrogels in Clinical Practices section establish the physical and biological conditions required for bioadhesive hydrogels to remain functional within complex chronic wound environments. However, stable adhesion, controlled transport, and mechanical compatibility do not merely support material retention; together, they create a persistent wound–material interface through which biochemical and mechanical signals can be locally transmitted to immune and tissue-resident cells. This intimate interface enables bioadhesive hydrogels to regulate immune and regenerative responses by controlling the presentation of therapeutic factors, inflammatory and oxidative cues, cell–material interactions, and mechanotransductive signaling (Figure 3d).

In this review, we define immunomodulation broadly to encompass both indirect actions, such as antioxidant and antibacterial effects that reduce inflammatory stimuli, and direct regulation of immune cell behavior, including macrophage polarization and cytokine remodeling, as illustrated in Figure 3d. Accordingly, hydrogel-mediated immunomodulation should be understood as an interfacial process arising from the coordinated regulation of oxidative stress, soluble-factor signaling, cellular adhesion, and mechanically induced responses. These mechanisms do not operate in isolation but converge at the wound–material interface to reshape the chronic wound microenvironment, attenuate persistent inflammatory activation, and facilitate the transition toward tissue regeneration.

At the biochemical level, one of the principal immunomodulatory mechanisms is the regulation of excessive oxidative stress. In chronic wounds, sustained production of ROS damages cellular membranes, proteins, nucleic acids, and ECM components. Morgan *et al*. reported that excessive ROS amplifies inflammatory signaling through activation of NF-κB and related redox-sensitive transcription factors, thereby increasing the expression of TNF-α, IL-1β, IL-6, and other pro-inflammatory mediators [66]. Bioadhesive hydrogels incorporating antioxidant polymers, polyphenols, catalytic nanoparticles, enzyme-mimetic materials, or ROS-responsive chemical linkages have been reported to reduce local ROS accumulation and alleviate oxidative damage. Beyond direct ROS scavenging, these materials may also reinforce endogenous cellular antioxidant defenses. In parallel, activation of endogenous antioxidant responses, particularly the Nrf2/HO-1 pathway, has been proposed to enhance cellular resistance to oxidative stress and suppress prolonged inflammatory signaling [67, 68]. Through the combined scavenging of extracellular ROS and regulation of intracellular redox pathways, these materials have been suggested to restore a biochemical environment more favorable for immune resolution and tissue repair.

A second interfacial mechanism is the localized and sustained presentation of soluble immunoregulatory signals. Hydrogel networks protect anti-inflammatory drugs, cytokines, growth factors, nucleic acids, proteins, and extracellular vesicles from premature degradation while enabling their localized and sustained release. Chen *et al*. reported that IL-4/IL-13 signaling activates STAT6-dependent macrophage responses associated with tissue repair, whereas IL-10 engages STAT3-related signaling through the activation of the IL-10 receptor [10]. Because these signals are retained near the adherent wound interface, their actions can extend beyond macrophage regulation to coordinated interactions among fibroblasts, endothelial cells, keratinocytes, and other wound-resident cells. Bioadhesive hydrogels have therefore been proposed to function as localized signaling reservoirs that coordinate macrophage reprogramming with re-epithelialization, angiogenesis, and ECM deposition.

In parallel with these biochemical effects, the physical characteristics of the adherent hydrogel interface can directly influence cellular behavior through mechanotransduction. Hydrogel stiffness, viscoelasticity, adhesive strength, surface microstructure, and ligand density have been reported to regulate integrin-mediated cell attachment, focal adhesion assembly, and cytoskeletal organization. Di and colleagues, and Panciera *et al*., reported that engagement of integrins, including αvβ3 and α5β1, induces focal adhesion kinase (FAK) phosphorylation and activates downstream Src and RhoA/ROCK signaling [69, 70]. These pathways have been reported to regulate actomyosin contractility, cytoskeletal tension, nuclear deformation, and cell migration. The resulting cytoskeletal remodeling can also alter the intracellular localization and transcriptional activity of YAP/TAZ, thereby connecting interfacial mechanics to cell proliferation, inflammatory gene expression, lineage-specific differentiation, and matrix production.

Mechanosensitive ion channels further translate forces generated at the wound–hydrogel interface into intracellular immune signals. Tang *et al*. reported that Piezo1 converts interfacial forces, tissue deformation, and matrix stiffness into Ca^2+^ influx and intracellular calcium waves in immune cells [71]. These calcium signals subsequently regulate enzymes and transcription factors involved in immune-cell activation, migration, and inflammatory mediator production. Crosstalk between Piezo1-mediated calcium signaling and redox-regulatory pathways, including Keap1–Nrf2 signaling, has been proposed to further connect hydrogel mechanics with cellular oxidative-stress responses. Hydrogel mechanics should therefore be regarded not merely as a structural parameter but as a biological input that determines how immune and regenerative cells sense and respond to the wound environment.

Together, these biochemical and mechanotransductive respon-ses are mediated through an interconnected signaling network rather than through a single dominant pathway. The NF-κB, STAT3/STAT6, Nrf2/HO-1, integrin–FAK, YAP/TAZ, and Piezo1-associated pathways illustrated in Figure 3d have been proposed as candidate signaling routes linking hydrogel properties with wound-healing responses [72]. These central routes interact with additional pathways, including TLR/MyD88 signaling, the NLRP3 inflammasome, MAPK and PI3K/Akt cascades, HIF-1α/VEGF-mediated hypoxic responses, and TGF-β/Smad-dependent matrix remodeling. Given the current state of evidence, the relative contribution of each pathway to hydrogel-mediated wound healing should be interpreted with appropriate caution, and future studies incorporating pathway-specific inhibitors, knockdown approaches, or reporter systems will be needed to establish direct causal links between hydrogel properties and these signaling routes. Moreover, the relative involvement of each pathway is likely to vary according to hydrogel composition and physical properties, incorporated therapeutic cargo, target cell type, infection status, and stage of wound healing.

Through their convergence at the material–tissue interface, these signaling mechanisms collectively promote the formation of a regenerative wound microenvironment. Suppression of oxidative and inflammatory signaling supports macrophage reprogramming, while controlled biochemical delivery and cell–material interactions promote fibroblast migration, endothelial activation, lineage-specific differentiation, angiogenic sprouting, and matrix deposition. The effectiveness of a bioadhesive hydrogel, therefore, depends not only on its ability to adhere to the wound but also on its capacity to couple stable interfacial contact with biologically instructive signaling.

These mechanistic principles provide the basis for classifying bioadhesive hydrogel systems according to how they establish and use the wound–material interface. Bioadhesive hydrogels for chronic wound management can be broadly organized into four complementary engineering strategies according to their principal design objective and mode of therapeutic action: (i) structural and protective hydrogels, (ii) bioactive and regenerative hydrogels, (iii) externally stimuli-responsive hydrogels, and (iv) sensing-integrated hydrogels. These categories are not mutually exclusive, and multifunctional platforms frequently combine characteristics from more than one strategy.

The first strategy, structural and protective bioadhesive hydrogels, primarily regulates inflammation by stabilizing the wound–material interface. Rapid conformal sealing and robust wet adhesion allow these materials to maintain continuous coverage of irregular, mobile, and fluid-rich wound surfaces. Their principal functions include mechanical protection, barrier formation, exudate and moisture management, and, where required, rapid hemostasis [46]. By reducing fluid leakage, microbial entry, tissue micromotion, and repeated disruption of the wound surface, these hydrogels indirectly alleviate persistent inflammatory stimulation and establish a stable microenvironment for subsequent tissue repair. Intimate contact with the wound bed also improves the local retention and effectiveness of endogenous signaling factors and subsequently delivered therapeutic agents. Thus, even in the absence of directly incorporated immunoregulatory components, structural stabilization can provide an important foundation for inflammation resolution.

Building on this stabilized interface, bioactive and regenerative bioadhesive hydrogels actively modify the biochemical and cellular wound environment. Therapeutic agents, including anti-inflammatory drugs, growth factors, proteins, nucleic acids, extracellular vesicles, metal ions, antioxidant compounds, and antimicrobial agents, can be incorporated into the hydrogel network for localized and sustained delivery. Living cells or beneficial microorganisms may also be encapsulated to provide continuous secretion of regenerative factors, oxygen generation, antimicrobial activity, or local immune regulation. Through these approaches, bioactive hydrogels can reduce excessive oxidative stress and inflammation, suppress microbial burden, improve tissue oxygenation, promote angiogenesis, and enhance re-epithelialization and ECM formation. Their therapeutic efficacy depends on the coordinated regulation of cargo stability, release kinetics, network degradation, and interactions with wound-resident cells.

Where temporal control is required, externally stimuli-respons-ive bioadhesive hydrogels allow therapeutic activity at the interface to be regulated by applied physical inputs. Electrical fields, light, heat, and ultrasound can be converted into localized biological, mechanical, or chemical responses at the hydrogel–tissue interface. Depending on the material design, these stimuli may regulate drug release, catalytic activity, ROS generation or scavenging, antimicrobial action, cell migration, angiogenesis, and immune-cell behavior. The combination of stable tissue adhesion with externally controlled activation enables therapeutic effects to be delivered at defined locations and time points. Treatment intensity and duration can consequently be adjusted as the wound environment evolves, for example, by prioritizing antimicrobial or anti-inflammatory activity during early treatment and regenerative stimulation during later phases.

Finally, sensing-integrated bioadhesive hydrogels extend the interface from a therapeutic platform into a source of wound-state information. These systems combine conformal wound coverage with the monitoring of physiological and biochemical parameters. Wound-associated variables such as pH, temperature, moisture, oxygenation, impedance, enzyme activity, metabolites, and inflammatory biomarkers can provide information regarding infection, inflammation, tissue viability, and healing progression. Integration of sensing elements with therapeutic components may enable treatment parameters to be adjusted according to the measured wound condition. Such platforms can link diagnostic information with controlled drug delivery, electrical stimulation, phototherapy, or other therapeutic interventions, thereby providing a foundation for adaptive or closed-loop wound management.

Although the four strategies differ in their primary design objectives, they act through overlapping and mutually reinforcing immunomodulatory mechanisms. Structural stabilization reduces external inflammatory stimuli and improves therapeutic retention; bioactive components directly regulate molecular and cellular signaling; externally activated systems provide spatial and temporal control over therapeutic activity; and sensing-integrated platforms support treatment selection and adjustment according to changes in the wound microenvironment. Their relative importance should therefore be tailored to wound etiology, infection status, exudate level, mechanical conditions, and healing stage.

Through this coordinated design, bioadhesive hydrogels can sequentially support wound sealing, inflammatory resolution, cell migration, angiogenic sprouting, re-epithelialization, ECM deposition, and tissue remodeling. They should therefore be regarded not simply as wound coverings but as multifunctional immunomodulatory interfaces that integrate physical stabilization, molecular transport, mechanobiological signaling, and temporally regulated therapeutic activity to promote controlled wound repair.

### Structural and protective bioadhesive hydrogels

**Figure 4:**
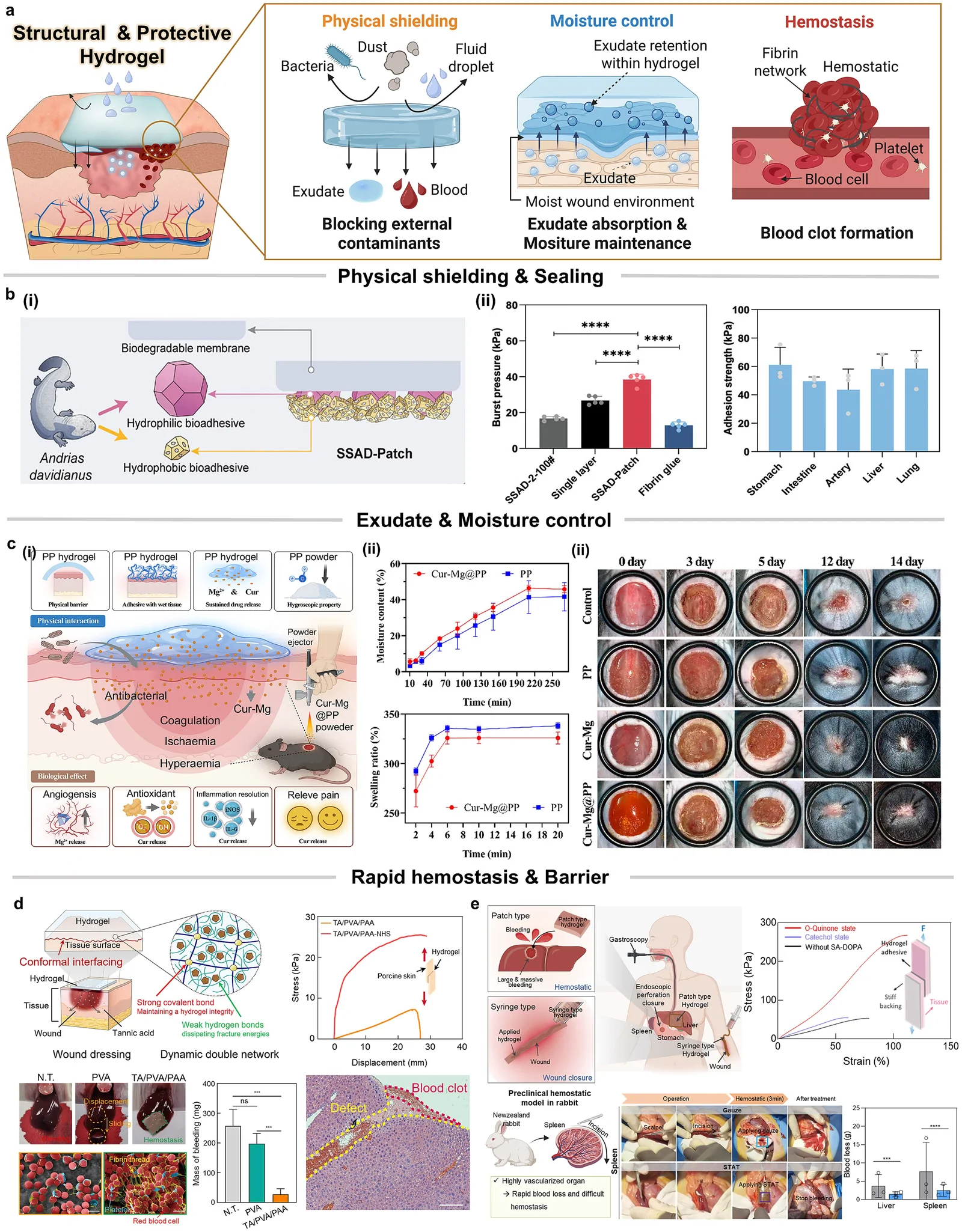
Structural and protective bioadhesive hydrogel. (**a**) Schematic of key functions of structural and protective bioadhesive hydrogel for early-stage wound-material interface stabilization. (**b**) The SSAD bioadhesive patch offers robust mechanics with effective shielding and sealing. Reproduced with permission [74]. Copyright 2024, Wiley-VCH GmbH. (**c**) Exudate and moisture management of the Cur-Mg@PP dressing. Adapted with permission [76]. Copyright 2023, American Chemical Society. (**d**) Rapid hemostasis and barrier function of the TA/PVA/PAA dynamic double-network hydrogel [80]. Adapted under the terms of the CC BY 4.0 License. (**e**) Quinone-mediated STAT hydrogel for instant wet-tissue adhesion and hemostasis. Reproduced with permission [81]. Copyright 2026, Springer nature.

The early phase of wound treatment represents a critical window that largely determines the success of subsequent immunomodulatory and regenerative strategies [50]. During this stage, structural and protective hydrogels contribute by actively suppressing early inflammatory triggers, such as microbial colonization and oxidative stress, through their reported ROS scavenging and antibacterial activity, thereby limiting the persistent activation signals that would otherwise reinforce chronic inflammation. Immediately after injury, the wound site is characterized by bleeding, exudate accumulation, tissue disruption, and continuous mechanical perturbation, creating a highly unstable environment [20]. Under such conditions, the therapeutic efficacy of bioactive or pro-regenerative interventions is contingent upon the prior establishment of a stable tissue–material interface. In this context, structural and protective bioadhesive hydrogels have emerged as an effective design strategy aimed at physically stabilizing the wound–material interface during the early stage of treatment and creating a regeneration-permissive microenvironment (Figure 4a) [73–81].

From an interfacial perspective, the early wound surface is wet, chemically heterogeneous, topographically irregular, and mechanically dynamic. Interfacial failure is therefore often driven by residual fluid, continuous motion, and insufficient initial bonding, rather than by inadequate bulk mechanical strength [73]. Accordingly, these bioadhesive hydrogels aim to establish a regeneration-permissive microenvironment using a soft, tissue-conformable platform [51]. This is achieved by integrating three coupled functions: protective barrier formation, moisture and exudate management, and rapid hemostasis under bleeding conditions.

Within this framework, conformal physical sealing plays a central role in protecting the wound by filling micro-scale voids and redistributing mechanical stress to prevent interfacial delamination [73]. At the same time, maintaining a balanced moist environment through active exudate management ensures optimal tissue compliance and prevents both excessive gel swelling and interfacial dehydration [50]. Furthermore, in bleeding scenarios, these hydrogels promote rapid hemostasis by restricting blood outflow and stabilizing clot formation [73]. By synergistically combining these functions, structural bioadhesives provide a robust foundation for subsequent tissue repair.

#### Physical shielding and sealing

Effective physical sealing in wet tissues often fails due to four coupled factors. Residual interfacial water and biological fluids prevent intimate contact and create leakage pathways. Dynamic and contaminated surface conditions, such as roughness, porosity, and continuous bleeding or exudation, further hinder sealing. Mechanically demanding loading modes are dominated by peeling and mixed-mode stresses. In addition, a mechanical mismatch between sealant and soft tissues promotes stress concentration, irritation, and debonding. Accordingly, reliable wet sealing requires rapid conformal contact and active fluid management to suppress microleakage. It also requires sufficient interfacial toughness and fatigue resistance to maintain structural integrity under swelling, friction, and repeated physiological deformation. To address these challenges, patch-type multilayer bioadhesive architectures have been explored to manage interfacial fluids in a stepwise manner. Liu *et al*. developed a multilayer patch derived from the skin secretions of *Andrias davidianus*, termed the SSAD Patch, integrating a microporous hydrophobic layer, a rapidly gelling hydrophilic layer, and a mechanically supportive biodegradable membrane (Figure 4b(i)) [74]. Upon contact, the hydrophobic layer suppresses early fluid ingress by trapping air in micropores. Under applied pressure, the interface transitions from a Cassie state to a Wenzel state. This transition releases trapped air and displaced interfacial water to increase effective contact, with residual defects largely eliminated at ~6 kPa. Subsequently, the hydrophilic fraction absorbs remaining moisture and gels within ~10 s, reinforcing adhesion through multivalent interactions. In a collagen sheet defect model, the SSAD Patch achieved a burst pressure of 38.4 ± 3.0 kPa, outperforming single-layer controls and fibrin glue (Figure 4b(ii)), consistent with membrane-enabled mechanical stabilization of the hydrated adhesive layer. The patch also exhibited robust wet adhesion across multiple organs and enabled rapid sutureless sealing in an *in vivo* intestinal defect model with reduced unwanted adhesion and inflammation relative to sutures.

Complementing passive multilayer shielding, press-actuated patches have been designed to actively repel blood and create a sufficiently dehydrated interface on demand. Yuan *et al*. reported a press-to-seal hydrogel patch containing surface-embedded press-actuated silicone oil micro reservoirs within a gelatin and poly(acrylic acid) matrix, enabling user-initiated and spatiotemporally controllable silicone oil release and area programmable blood repulsion [75]. Upon pressing, crushed micro reservoirs release silicone oil to repel blood and water and induce interfacial dehydration for tight hydrogel tissue anchoring, while multivalent physical interactions, including hydrogen bonding and electrostatic coupling, contribute to wet adhesion. Sealing performance was validated by burst pressure testing, where the patch reached 200.0 ± 4.3 mmHg, exceeding normal systolic blood pressure, and outperforming commercial n-butyl cyanoacrylate and a control hydrogel. The study also highlighted a design window in which insufficient oil limits dewetting, whereas excessive oil can remain at the interface and impede adhesion, underscoring the need for tunable fluid management under bleeding conditions.

#### Exudate and moisture control

Effective exudate management and maintenance of a moist yet stable wound interface are essential for rapid re-epithelialization while also reducing periwound maceration and infection risk in fluid-rich wounds. To address this need, Gong *et al*. developed an exudate-responsive burn dressing, Cur-Mg@PP, consisting of an ionically crosslinked ε-PLL/γ-PGA (PP) matrix integrated with a curcumin-magnesium polyphenol network (Cur-Mg) [76]. Applied as a dry powder directly to the wound, Cur-Mg@PP rapidly absorbs exudate and converts into a wet-adhesive hydrogel, enabling conformal sealing and barrier protection while supporting sustained local delivery of Mg^2+^ and curcumin. Quantitative characterization confirms fast moisture handling (Figure 4c(i)). Under high humidity, both PP and Cur-Mg@PP show rapid moisture uptake that plateaus at ~220 min, with Cur-Mg@PP slightly higher. In aqueous swelling tests, both materials hydrate within minutes. They reach a swelling ratio of ~326% (Cur-Mg@PP) versus ~338% (PP) at 20 min (Figure 4c(ii)). This suggests that Cur-Mg incorporation modestly tightens the ionic network while retaining strong exudate absorption. Beyond regulating fluid quantity, Cur-Mg@PP also improves the “quality” of the trapped exudate. It provides broad-spectrum antibacterial activity and delivers combined antioxidant/anti-inflammatory, pro-angiogenic, and analgesic effects via sustained Cur-Mg/Mg^2+^ release. *In vivo*, serial wound images show accelerated burn closure and improved remodeling in the Cur-Mg@PP group compared with control, PP, and Cur-Mg alone (Figure 4c(iii)). Healing approaches near-completion by Days 12–14. Overall, Cur-Mg@PP demonstrates an absorb-and-seal strategy that couples rapid exudate capture and stable moist coverage with local antimicrobial and regenerative microenvironment regulation.

Similarly, Liu *et al*. proposed a double-network (DN) hydrogel composed of poly(vinyl alcohol) (PVA), agarose, hyperbranched polylysine (HBPL), and tannic acid (TA) (hereafter denoted as PAHT), which shifts exudate management from passive absorption to active microenvironment regulation [77]. The hydrogel combines a crystallized PVA primary physical network with a hydrogen-bonded agarose secondary network, which mitigates the excessive swelling and mechanical weakening often observed in conventional single-network gels. As a result, PAHT exhibits controlled swelling and reaches an equilibrium of ~61.1% within 10 h, helping maintain barrier function and dimensional stability over the wound. The incorporation of hyperbranched polylysine (HBPL) and tannic acid (TA) further reinforces the matrix, enabling a fracture stress of 0.57 MPa even in the fully swollen state, and supports hydration-dependent diffusion-controlled release for sustained antimicrobial and antioxidant effects within absorbed exudate.

From a moisture-management perspective, regulating the biochemical “quality” of the trapped fluid is as important as controlling its quantity. In PAHT, the moisture-retentive hydrogel matrix helps maintain prolonged contact between the bioactive components and the absorbed exudate, thereby enabling an *in situ* “cleansing” function within the dressing. Specifically, HBPL/TA-mediated antibacterial activity and ROS scavenging at the material-exudate interface can reduce local inflammatory triggers in the retained fluid and help protect adjacent healthy tissue from secondary damage. This biochemical regulation within a stable, moist environment may also support more orderly ECM remodeling (e.g. collagen deposition) during healing. Ultimately, the synergy between structural toughness and active exudate regulation helps ensure that regeneration proceeds under stable physical and chemical conditions, offering a comprehensive strategy for managing fluid-rich wound environments.

However, interpreting these swelling capacities requires caution, as an optimal swelling range for highly exuding chronic wounds remains undefined [78]. Highly exuding chronic wounds generate 0.5–1.0 ml exudate per 10 cm^2^ daily (up to 0.17–0.63 g/cm^2^/day), necessitating quantitative hydrogel design [45]. Furthermore, chronic exudate contains MMP-9 and neutrophil elastase that degrade polymer networks, compromising long-term mechanical integrity and wet adhesion [79]. These enzyme-induced alterations often impair the barrier function and drug-release kinetics of hydrogels. Because rate-matched fluid handling remains rarely evaluated, optimal swelling ranges are undefined. Engineering enzyme-tolerant hydrogels matched to specific exudate production rates thus represents a critical avenue for translating these bioadhesive systems into clinical therapeutics.

#### Rapid hemostasis and barrier

A paramount challenge in wound care is achieving instantaneous hemostasis, particularly in emergency scenarios where high-pressure hemorrhage can lead to fatal exsanguination. Standard clinical protocols, such as mechanical sutures or conventional pressure-sensitive dressings, often fail to establish an immediate, leak-proof seal on actively bleeding soft tissues, especially under continuous physiological motion. Consequently, there is an urgent clinical need for multifunctional bioadhesives that can robustly adhere to wet, dynamic tissue surfaces while simultaneously accelerating the intrinsic coagulation cascade to stabilize wounds under harsh physiological conditions.

As shown in Figure 4d, Park *et al*. developed a tannic-acid-incorporated poly(vinyl alcohol)/poly(acrylic acid) double-network hydrogel (PVA/TA/PAA) to couple rapid sealing with procoagulant activity [80]. Through EDC/NHS activation, the PAA network forms covalent amid bonds with tissue amines, yielding a lap shear strength of ~35.98 kPa, while TA facilitates blood clotting to achieve stable hemostasis within ~10 s in a mouse liver model. However, chemical activation steps like EDC/NHS often require lengthy preparation times that limit their utility in acute emergencies. To overcome this, Kim *et al*. developed the STAT (Strong Tissue-adhesive And Thrombostatic) hydrogel, which achieves instant wet-tissue adhesion without exogenous activators (Figure 4e) [81]. The STAT hydrogel features a primary network composed of PVA, dopamine-functionalized sodium alginate (SA-DOPA), TA, and sodium tetraborate decahydrate (borax), which is interpenetrated with a PAA secondary network. Simple mechanical kneading partially oxidizes the catechol groups of SA-DOPA into reactive *o*-quinone species, enabling immediate covalent bonding with tissue amines upon contact. Synergizing this instant adhesion with TA-driven platelet activation, the STAT hydrogel rapidly halted bleeding within seconds in both mouse liver puncture and severe rabbit spleen and liver hemorrhage models.

Table 2 also includes *in situ*-gelling adhesives that prioritize rapid deployment and on-demand solidification directly at the bleeding site, which is particularly relevant for minimally invasive or endoscopic hemostasis [82–86]. To systematically evaluate these platforms, Table 2 summarizes their material composition, gelation time, wet adhesion strength, immunomodulatory outcome, wound healing model, and translational considerations. To achieve reliable hemostasis in dynamic environments, these bioadhesives exhibit a wide range of wet adhesion strengths, spanning from 5.29 to 35.98 kPa, depending on their specific crosslinking mechanisms. Furthermore, they actively provide immunomodulatory outcomes, such as ROS-scavenging and antibacterial properties, to facilitate early wound repair. For instance, Xia *et al*. reported the HACN-PEG ultrafast-gelling adhesive hemostatic hydrogel for acute upper gastrointestinal hemorrhage, designed for endoscopic delivery [82]. HACN-PEG combines a hyaluronic-acid-based HACN network formed via thiourea–catechol coupling with a second network formed from PEGSH through disulfide bonding, enabling rapid network formation and wet adhesion. The reported gelation time is 1.7 s, and the lap shear strength is ~12.5 kPa, with hemostasis achieved in 59 s in a rat femoral artery model. Furthermore, in a porcine upper gastrointestinal hemorrhage model, endoscopic catheter delivery of the hydrogel achieved hemostasis in ~2.1 min and maintained adhesion for 48 h, highlighting its strong translational potential.

**Table 2:** Rapid-hemostasis and barrier bioadhesive hydrogels for wound healing

Material (Composition & Key feature)	Gelation time	Wet adhesion strength	Immunomodulatory outcome	Hemostasis model & Result	Wound healing model & Key result	Translational considerations	Refs.
TA/PVA/PAA hydrogel (tannic acid/PVA/polyacrylic acid; NHS-activated)	N/A (pre-formed patch)	35.98 kPa (NHS-activated)	No cytokine/IHC data F4/80: comparable to sham (no induced inflammation)	Mouse liver puncture: ~10 s	Not evaluated (hemostasis-focused patch)	On-demand removal (NaHCO₃/glutathione) Non-swellable Antibacterial (*E. coli*/*S. aureus*)	[80]
HACN-PEG hydrogel (HA-catechol/thiourea + PEGSH disulfide)	1.7 s	~12.5 kPa (chicken skin)	Mild–moderate inflammation (MPO/IL-1β expression)	Rat femoral artery: 59 ± 7.9 s Porcine endoscopic GI: ~2.1 min	Not evaluated (endoscopic hemostatic sealant)	Endoscopic catheter delivery Validated in the porcine model 48 h *in situ* retention	[82]
CSG-PEG/DMA/Zn hydrogel (PEG-chitosan + methacrylamide-dopamine + Zn^2+^, photo-crosslinked)	~15 s (UV 365 nm)	11.5 kPa	CD68↓, CD31↑ *vs* Tegaderm™ (P < 0.05)	Mouse liver: 223.7 *vs* blank 689.2 mg Tail: 33.4 vs blank 237.8 mg (P < 0.01)	MRSA full-thickness wound Day 7: 86% *vs* 63–74% comparators Day 14: > 95% closure	*In-situ* UV crosslinking L929 cytocompatible (> 5 d) ~20% residual mass at Day 13	[83]
CaGOD/ZnO hydrogel (caffeic-acid-gelatin + oxidized dextran, Schiff-base + Zn^2+^-catechol)	~30 s	26.9 ± 3.1 kPa	Sustained Zn^2+^ release Antibacterial/anti-inflammatory	Rat liver incision: ~15 s Ventricular perforation: ~20 s	MRSA full-thickness wound Day 14: 99% *vs* 78.3% (PBS)	EDTA on-demand removal Designed for field first-aid use	[84]
QCS/TA2.5 Hydrogel (quaternized chitosan + tannic acid dynamic network)	13.3 ± 0.7 min	~35 kPa (graph-estimate)	IL-6↓/IL-4↑ (immunofluorescence) VEGF/PCNA↑	22–250 s across liver/tail/femoral-artery models	Full-thickness wound Day 14: 92.8% *vs* 81.6% (Tegaderm™)	Low-cost, natural polyphenol crosslinker No on-demand removal system	[85]
DNAgel (salmon-sperm DNA + PEGDA, NETs-inspired)	N/A (pre-formed; 37°C/1 h manufacture)	5.29 ± 0.80 kPa	Qualitative only (H&E) Fewer neutrophils *vs* GS at Day 7	Liver: 0.66 g *vs* GS 2.07 g *vs* control 5.87 g Tail: 87.7% less blood loss *vs* GS	Day 10: 99.33% closure Hair follicles/sebaceous glands regenerated	Biodegrades in 2–3 days No second extraction needed Field-deployable (pre-formed)	[86]

*CSG* chitosan graft, *PEG* polyethylene glycol, *DMA* dopamine methacrylamide, *HACN* hyaluronic acid–catechol–thiourea derivative, *HA* hyaluronic acid, *PEGSH* thiol-terminated polyethylene glycol, *TA* tannic acid, *PVA* poly(vinyl alcohol), *PAA* poly(acrylic acid), *NHS* N-hydroxysuccinimide, *UV* ultraviolet, *MPO* myeloperoxidase, *IL* interleukin, *IHC* immunohistochemistry, *GI* gastrointestinal, *MRSA* methicillin-resistant S. aureus, *PEGDA* polyethylene glycol diacrylate, *NETs* neutrophil extracellular traps, *QCS* quaternized chitosan, *H&E* hematoxylin and eosin, *VEGF* vascular endothelial growth factor, *PCNA* proliferating cell nuclear antigen, *PBS* phosphate-buffered saline

A second contrasting example is the CSG-PEG/DMA/Zn photo-crosslinked multifunctional composite hydrogel wound dressing reported by Yang *et al*. [83]. In CSG-PEG/DMA/Zn, methacrylate-functionalized chitosan grafted with PEG forms a covalently crosslinked network via photo-initiated polymerization, while methacrylamide dopamine introduces catechol groups that enhance wet adhesion and provide antioxidant activity. Zinc ions coordinate with catechol groups, contributing antibacterial functionality and additional reversible crosslinking interactions. This hydrogel forms rapidly under ultraviolet irradiation, with a gelation time of ~15 s, and exhibits a lap shear strength of 11.5 kPa, illustrating a design that prioritizes rapid *in situ* formation and multifunctionality, albeit with more modest interfacial shear strength than strongly covalently anchored patch sealants.

In addition to the representative systems discussed above, Table 2 also includes several “first-aid-oriented” adhesive hemostatic hydrogels that emphasize robust performance in bloody, mobile, and infection-prone environments. Cheng *et al*. developed a mussel-inspired, multi-crosslinked CaGOD/ZnO hydrogel dressing formed via reversible Schiff-base linkages between caffeic-acid-modified gelatin (CaG) and oxidized dextran (ODex), together with Zn^2+^-catechol coordination [84]. The incorporation of ZnO not only reinforced cohesion and accelerated gelation but also enabled sustained Zn^2+^ release for broad-spectrum antibacterial effects and coagulation support. Correspondingly, the hydrogel showed markedly shortened clotting time *in vitro* (≈2.9 min) and rapid hemostasis in severe bleeding models (≈15 s in rat liver incision and ≈20 s in ventricular perforation) while also improving MRSA-infected full-thickness wound healing. Together with other tabled examples such as QCS/TA dynamic physical networks and NETs-inspired DNA hydrogels, these studies highlight a convergent design logic for next-generation emergency hemostatic dressings [85, 86]. They couple wet adhesion and mechanically resilient, reversible crosslinking with antimicrobial/antioxidant functionality and coagulation-promoting cues.

Collectively, these examples indicate that a wide range of gelation times, from 1.7 s to pre-cured patches requiring 1 h of fabrication, is acceptable because the optimal kinetics are dictated by the clinical delivery route. *In situ* adhesives require ultrafast gelation to prevent washout in actively bleeding environments, whereas for pre-formed patches, gelation time is merely an *ex vivo* manufacturing parameter. Furthermore, hemostatic performance cannot be uniformly predicted by gelation kinetics alone due to the inherent anatomical and physiological differences across animal models. For example, the same 1.7-s gelation of HACN-PEG resulted in 59-s hemostasis in a rat model but required ~2.1 min in a porcine model. Such distinctions highlight the necessity of evaluating hemostatic bioadhesives within their specific target applications rather than through direct numerical comparisons. Ultimately, next-generation hemostatic bioadhesives should be engineered by jointly tuning gelation kinetics, wet interfacial adhesion, and network cohesion/mechanical resilience, and the optimal balance is application-specific. Patch-based systems such as PVA/TA/PAA(-NHS) prioritize durable tissue anchoring and instantaneous sealing, whereas *in situ*-gelling formulations (e.g. HACN-PEG, CSG-PEG/DMA/Zn, and CaGOD/ZnO) broaden the design space toward minimally invasive deployment and multifunctional wound management.

#### Cooling-assisted wound care

Beyond rapid hemostasis, an emerging consideration in wound management is the local thermal microenvironment. Acute tissue injury often induces localized hyperthermia due to inflammation and elevated metabolism, which can favor bacterial growth and prolong inflammation. To address this, Cao *et al*. demonstrated a bioelectronic platform integrating a passive radiative cooling (PRC) layer composed of electrospun PVDF-co-CTFE and PVDF fiber mats [87]. Designed to reject solar energy while dissipating metabolic heat, this platform provides an intelligent thermal shield for wounds under outdoor or emergency conditions.

Effective thermal conduction is facilitated by robust interfacial adhesion achieved via dynamic hydrogen bonding within a liquid metal (LM)-PVA composite, eliminating insulating air gaps even under dynamic deformation. In a mouse wound model, integrating radiative cooling with electrical stimulation significantly accelerated tissue regeneration, yielding superior wound closure and enhanced collagen deposition compared with commercial bandages. These results indicate that cooling functionality does not merely act as a passive shield but actively optimizes the thermal microenvironment to promote rapid healing.

Despite these significant advancements in structural and protective bioadhesives, several critical challenges persist. First, the inherent trade-off between rapid gelation kinetics and high interfacial toughness remains a primary bottleneck; systems that bond instantly often lack the long-term fatigue resistance required for highly mobile anatomical sites. Second, while multilayer and composite architectures offer superior sealing, their increased structural complexity may pose challenges for large-scale standardized manufacturing and clinical ease of use. Furthermore, chronic and high-volume exudation can lead to hydrogel over-swelling and subsequent delamination. Consequently, further optimization of swelling pressure and cross-linking density is required to maintain stable adhesion under these conditions. Future designs must therefore focus on achieving a “dynamic equilibrium” where the hydrogel maintains both its hermetic seal and mechanical compliance throughout the entire duration of the healing process, regardless of fluctuating moisture levels.

### Bioactive and regenerative bioadhesive hydrogels

The fundamental limitation of conventional wound dressings lies in their passive nature, which merely provides a physical barrier without addressing the complex biochemical imbalances of the wound microenvironment. Although structural stabilization establishes a protected wound interface, it is often insufficient to resolve persistent inflammation, hypoxia, microbial burden, and impaired regenerative signaling. Bioactive and regenerative hydrogels, therefore, extend the function of bioadhesion beyond wound coverage by actively modifying the cellular and biochemical microenvironment.

**Figure 5:**
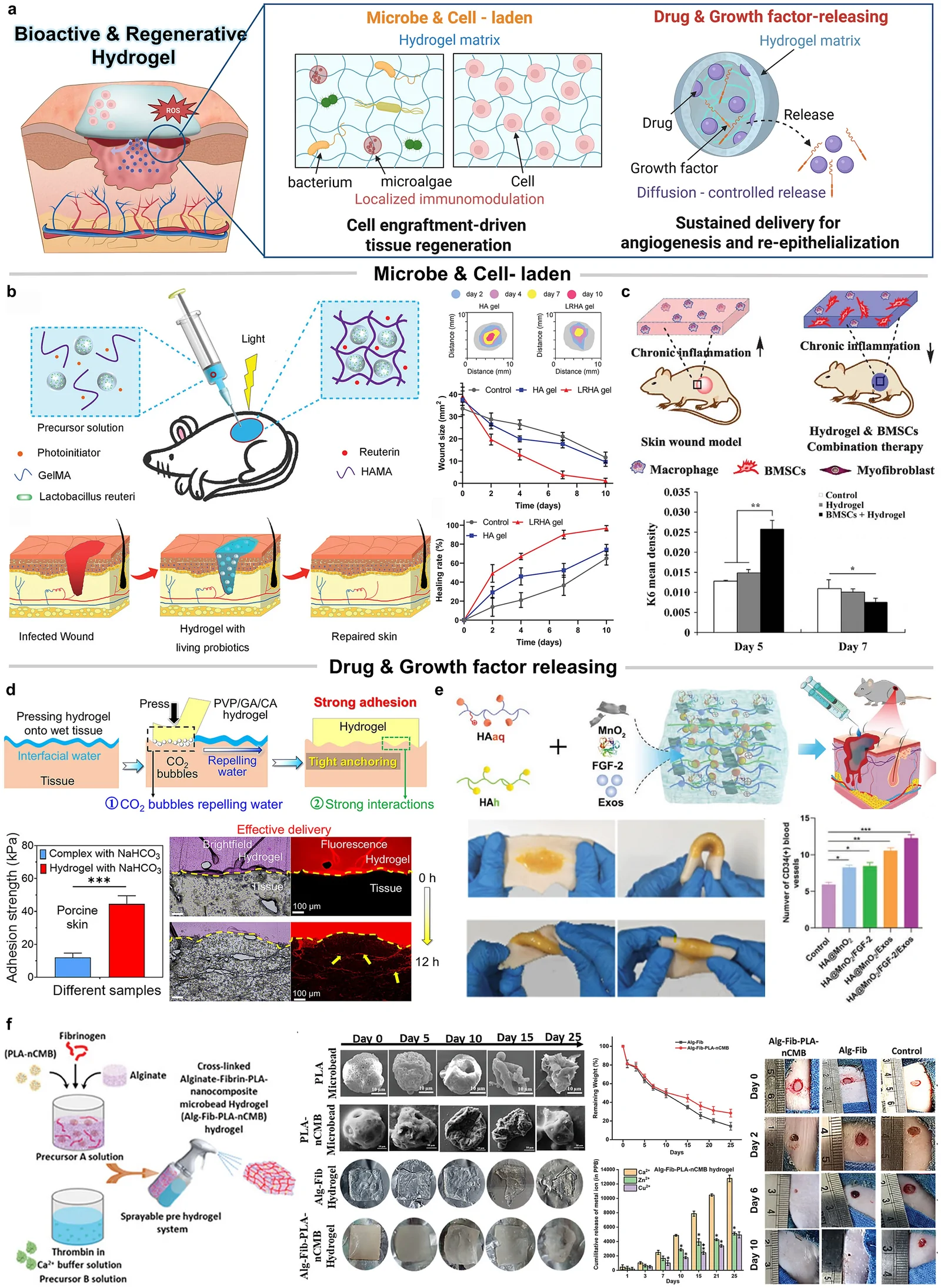
Bioactive and regenerative bioadhesive hydrogel. (**a**) Schematic of key functions of bioactive and regenerative bioadhesive hydrogel for early-stage wound-material interface stabilization. (**b**) In situ photocrosslinked living bacterial hydrogel for accelerated healing of infected wounds [93]. Adapted under the terms of the CC BY 4.0 License. (**c**) BMSCs-laden anti-inflammatory hydrogel for enhanced diabetic wound healing [95]. Reproduced under the terms of the CC BY 4.0 License. (**d**) CO₂-driven interfacial dehydration for enhanced GA/EGF delivery and macrophage polarization toward a reparative phenotype. Reproduced with permission [101]. Copyright 2025, Wiley-VCH GmbH. (**e**) All-in-one injectable HA@MnO₂/FGF-2/Exos hydrogel for accelerated healing of oxidative diabetic wounds. Reproduced with permission [104]. Copyright 2021, Small. (**f**) Time-staggered release of multi-metallic ions from a sprayable Alg-Fib-PLA-nCMB hydrogel for accelerated wound healing. Reproduced with permission [106]. Copyright 2024, Wiley-VCH GmbH

As illustrated in Figure 5a, these platforms can be broadly divided into two complementary strategies: the incorporation of living biological components and the controlled delivery of therapeutic factors. Microbe- and cell-laden hydrogels provide sustained biological activity through metabolite secretion, oxygen generation, immunomodulatory signaling, and regenerative cell–cell interactions. In parallel, drug- and growth-factor-releasing hydrogels function as localized reservoirs that protect therapeutic cargos and regulate their release within the wound bed. Together, these strategies support the resolution of inflammation, control of infection and hypoxia, angiogenesis, re-epithelialization, and tissue remodeling [88]. The following sections discuss the material architectures and therapeutic mechanisms underlying living-component integration and controlled molecular delivery in bioadhesive hydrogel systems.

#### Bioactive microbe and cell-laden hydrogels

The therapeutic management of chronic wounds requires bioactive composite platforms. These platforms should address persistent inflammation, impaired neovascularization with hypoxia, and susceptibility to infection. Recent wound biomaterials, therefore, combine a supportive hydrogel matrix with living components. The hydrogel conformally covers the defect and maintains hydration, while the living payload remodels the wound microenvironment. Unlike static materials, living-component-based hydrogels can continuously respond to local biological conditions through cell metabolism, paracrine signaling, and the secretion of bioactive molecules. Encapsulated mesenchymal stem cells, endothelial or skin-resident cells, probiotics, and photosynthetic microorganisms may provide sustained immunomodulatory factors, antimicrobial metabolites, angiogenic cues, or locally generated oxygen. Such continuous biological activity is particularly advantageous in ischemic, infected, or chronically inflamed wounds, where the therapeutic requirements evolve and cannot be fully addressed by a single preloaded dose [89–92].

The hydrogel matrix plays several roles beyond simple encapsulation. It provides a hydrated and mechanically supportive niche that preserves cell or microbial viability, facilitates the diffusion of oxygen, nutrients, metabolites, and secreted factors, and restricts uncontrolled dispersal of the living payload. When combined with stable tissue adhesion, these systems maintain living components close to the wound bed, thereby improving the local persistence and effectiveness of their therapeutic activity.

Microbe-laden hydrogels can directly reduce bioburden and modulate the local microbiological niche without relying solely on broad-spectrum antimicrobials. Ming *et al*. reported a living bacterial hydrogel dressing for accelerated healing of infected wounds (Figure 5b) [93]. The system encapsulated the probiotic *Lactobacillus reuteri* within GelMA hydrogel microspheres and further immobilized these microspheres in a photocrosslinked methacrylate-modified hyaluronic acid (HAMA) network. This hierarchical design supported bacterial viability and metabolite diffusion while reducing the risk of bacterial escape into surrounding tissue. Antibacterial activity was attributed to secreted metabolites such as lactic acid and reuterin, enabling inhibition of pathogens, including *Staphylococcus aureus*. *In vivo*, the *L. reuteri*-laden hydrogel (LRHA) showed faster wound size reduction and higher healing rates over time than cell-free hydrogel and untreated controls, consistent with improved histological repair.

Beyond single-strain approaches, microbial consortia embedded in hydrogels can address both hypoxia and infection simultaneously. Chen *et al*. developed a Living Microecological Hydrogel (LMH) based on a thermosensitive Pluronic F-127 matrix reinforced with polydopamine (PDA) [94]. The platform co-encapsulated photosynthetic algae (Chlorella) and probiotic bacteria (*Bacillus subtilis*), each loaded at ~10^7^ cells/ml. Chlorella generated dissolved oxygen through photosynthesis to alleviate local hypoxia and support microbial metabolism, while *B. subtilis* secreted antimicrobial lipopeptides that inhibited pathogens such as *S. aureus*. *In vivo*, the LMH group exhibited markedly improved wound closure and thicker regenerated tissue relative to controls.

In diabetic and chronic settings, restoring host regenerative signaling and immune balance is also critical. Chen *et al*. reported an anti-inflammatory hydrogel laden with bone marrow mesenchymal stem cells (BMSCs) at 10^6^ cells/ml to enhance healing in db/db mice (Figure 5c) [95]. The carrier was a thermosensitive PNIPAM-PAA hydrogel prepared by copolymerizing N-isopropylacrylamide (NIPAM) with a biodegradable multifunctional poly(amidoamine) (PAA) crosslinker. The design targeted the chronic M1-dominant inflammatory niche typical of diabetic wounds and supported a shift toward M2 polarization and sustained paracrine signaling. Re-epithelialization was assessed using Keratin 6 (K6), where the K6 mean density reached ~0.026 on Day 5, approximately two-fold higher than the ~0.013 observed in controls.

For topographically complex defects, bioprinted multi-cell constructs can provide structured tissue replacement and guide organized regeneration. Wu *et al*. reported planar and curvilinear bioprinted tri-cell-laden hydrogel skin substitutes using a polyurethane (PU)-gelatin composite bioink. Polyurethane provided elasticity, printability, and mechanical stability, while gelatin contributed ECM-mimetic and cell-adhesive features [90]. After printing, Ca^2+^-mediated crosslinking improved handling and shape fidelity. The constructs incorporated keratinocytes, fibroblasts, and endothelial progenitor cells (EPCs) to support epidermal restoration, dermal matrix formation, and neovascularization, respectively.

These studies indicate that chronic-wound hydrogels can benefit from living payloads through complementary mechanisms. Microbe-laden systems can suppress pathogens via secreted metabolites. Microecological consortia can improve oxygenation while providing antimicrobial defense. Stem-cell-laden hydrogels can reprogram inflammatory niches and preserve pro-regenerative signaling. In addition, multi-cell bioprinted constructs can offer structured support for barrier restoration and vascularization in complex defects. Together, microbe- and cell-laden living hydrogels provide convergent routes to re-enable re-epithelialization, angiogenesis, and dermal reconstruction in nonhealing wounds.

#### Controlled delivery of therapeutic factors

The therapeutic efficacy of hydrogels in chronic wound management depends on their ability to provide a controlled and localized supply of bioactive agents. In the hostile microenvironment of a chronic wound, fragile therapeutic cargos such as growth factors, proteins, and exosomes are highly susceptible to rapid enzymatic degradation by proteases [96]. Hydrogels are indispensable as delivery systems because their highly hydrated, porous networks provide a protective physical shield that preserves the biological integrity of these sensitive molecules. Furthermore, the tunable cross-linking density of hydrogels allows for the tunable diffusion control, enabling a sustained release profile that maintains therapeutic concentrations over extended periods. This capability is crucial for overcoming the limitations of conventional topical treatments, which often require frequent applications due to rapid drug loss. When integrated with bioadhesion, controlled-delivery hydrogels maintain intimate contact with the wound bed and reduce the loss of therapeutic agents through leakage, dilution, or dressing displacement [97]. This localized retention is particularly important for processes such as macrophage reprogramming, angiogenesis, fibroblast activation, and re-epithelialization, which require appropriate concentrations and timing of biochemical signals rather than brief exposure to a single therapeutic dose [98–100].

Depending on the intended application, therapeutic release may be governed by passive diffusion, hydrogel degradation, reversible molecular interactions, or responsiveness to wound-associated cues such as pH, ROS, glucose, and enzymatic activity. Hydrogels can therefore function not only as passive reservoirs but also as programmable delivery interfaces that coordinate anti-inflammatory, antimicrobial, pro-angiogenic, and regenerative signals with the changing wound environment. By serving as an active reservoir, hydrogels support sustained regenerative signaling beyond initial wound coverage.

Interfacial water management via gas-driven anchoring significantly enhances the delivery efficiency of growth factors and small molecules in wet environments. As demonstrated by He *et al*. in Figure 5d, the polyvinylpyrrolidone/gallic acid/citric acid (PVP/GA/CA) hydrogel generates CO₂ bubbles in situ via a sodium bicarbonate (NaHCO₃) reaction, which repels interfacial water [101]. This mechanism enables robust wet adhesion, reaching 44.5 kPa on porcine skin. This system delivers GA and Epidermal Growth Factor (EGF) directly into the wound bed. This effective delivery mechanism prevents the dilution of these therapeutic agents by wound exudates, which is a common failure point in traditional treatments. Quantitative analysis showed that this targeted delivery led to a 16.6-fold reduction in bacterial density and a dramatic shift in macrophage polarization, with 42.8-fold fewer M1 macrophages and 43.8-fold more M2 macrophages compared to commercial gels. Furthermore, fluorescence imaging at 12 h confirms the deep penetration of GA and EGF into the tissue, stimulating angiogenesis and cell proliferation as evidenced by a 4.7-fold increase in CD31 density and 3.3-fold more PCNA-positive cells.

Complementary to interfacial water control, adhesive hydrogel patches can further improve local drug retention on wet skin while mitigating burst release. In addition, microenvironment-responsive networks can tune release timing to match pathological wound conditions. Kim *et al*. developed an adhesive double-network PAM/PDA hydrogel patch. PAM forms the primary network, while PDA provides catechol-mediated tissue adhesion [102]. Extra-large-pore mesoporous silica nanoparticles (XL-MSNs) were embedded as drug reservoirs and loaded with dexamethasone. This design reduced burst release compared with direct drug loading in the gel, improving local retention and alleviating inflammatory hallmarks in an *in vivo* dermatitis model. Extending this concept to disease-adaptive delivery, Wu *et al*. designed an injectable, self-healing cellulose-based composite hydrogel (POMC/PVA) [103]. The network is crosslinked via dynamic imine and boronate ester bonds. It preferentially degrades under pH/ROS conditions characteristic of diabetic infected wounds, enabling staged release. Mesoporous ZnO (mZnO) is released early for antibacterial and anti-inflammatory effects. Recombinant human type I collagen (rhCOL1) is then released more slowly to promote angiogenesis and tissue regeneration.

Building on cue-responsive release, catalytic microenvironment regulation combined with timed-release strategies offers a sophisticated approach to diabetic wound healing. Xiong *et al*. developed an *in situ* injectable hyaluronic acid (HA) hydrogel incorporating manganese dioxide (MnO₂), fibroblast growth factor-2 (FGF-2), and M2 macrophage-derived exosomes (M2-Exos), termed HA@MnO₂/FGF-2/Exos (Figure 5e) [104]. The hydrogel serves as a dual-delivery vehicle for FGF-2 and exosomes. This platform integrates MnO₂ nanosheets to convert excess H₂O₂ into O₂, simultaneously mitigating oxidative stress and providing a platform for the sequential release of these biological cues. The release kinetics are precisely tuned so that 34%–45% of encapsulated FGF-2 is released within 24 h and reaches 75% by Day 7, while the M2-derived exosomes are steadily discharged over 21 days. This timed-release profile ensures a sustained angiogenic stimulus throughout the various stages of healing. The biological impact is highlighted by the significant recruitment of CD31(+) and CD34(+) vessels, along with enhanced blood perfusion, which facilitates the transition from the inflammatory phase to tissue remodeling in recalcitrant diabetic ulcers. Stimuli-responsive, particle-assisted delivery systems further enable on-demand release of growth factors while providing ancillary antibacterial functions. Siebert *et al*. reported a 3D-printed GelMA-based hydrogel scaffold incorporating tetrapodal ZnO (t-ZnO) microparticles whose surfaces were H₂O₂-treated to enhance protein adsorption [105]. VEGF was immobilized on the t-ZnO and could be released on demand under visible green light, while the ZnO component simultaneously contributed intrinsic antibacterial activity. The printed porous architecture maintained a hydrated environment and supported localized growth-factor delivery, resulting in enhanced angiogenesis and accelerated wound closure *in vivo*.

Sprayable interpenetrating network hydrogels reinforced with polymeric microcarriers provide a versatile platform for the long-term delivery of inorganic therapeutics. As shown by Ghosal *et al*. (Figure 5f), the Alg-Fib-PLA-nCMB formulation comprises an alginate (Alg) and fibrin (Fib) hydrogel network infused with polylactic acid (PLA) microcarriers [106]. These microcarriers encapsulate Ca-, Cu-, and Zn- containing nanoparticles and enable controlled release of therapeutic metal ions. The system exhibits a steady hydrolytic degradation profile over 25 days and releases ions within therapeutic ranges, including Ca^2+^ up to 12.76 ppm, Cu^2+^ up to 4.90 ppm, and Zn^2+^ up to 5.11 ppm. This sustained release aligns with the prolonged temporal requirements of wound repair and is associated with a 55% 2,2-diphenyl-1-picrylhydrazyl (DPPH) radical scavenging rate and enhanced antibacterial activity. *In vitro*, the synergistic delivery of these ions through the alginate–fibrin matrix increased collagen I deposition by 42.5% and CD31 expression by 49%. *In vivo*, the platform achieved complete wound closure within 6 days and outperformed commercial formulations that required 12 days to reach comparable outcomes. Through these diverse strategies, ranging from CO₂-mediated anchoring to timed exosome release and ion-loaded microcarriers, bioadhesive hydrogels have evolved into active therapeutic devices. These platforms not only ensure the structural closure of the wound but also orchestrate the complex molecular signaling required for functional tissue regeneration.

### External-stimuli-based therapeutic bioadhesive hydrogels

**Figure 6:**
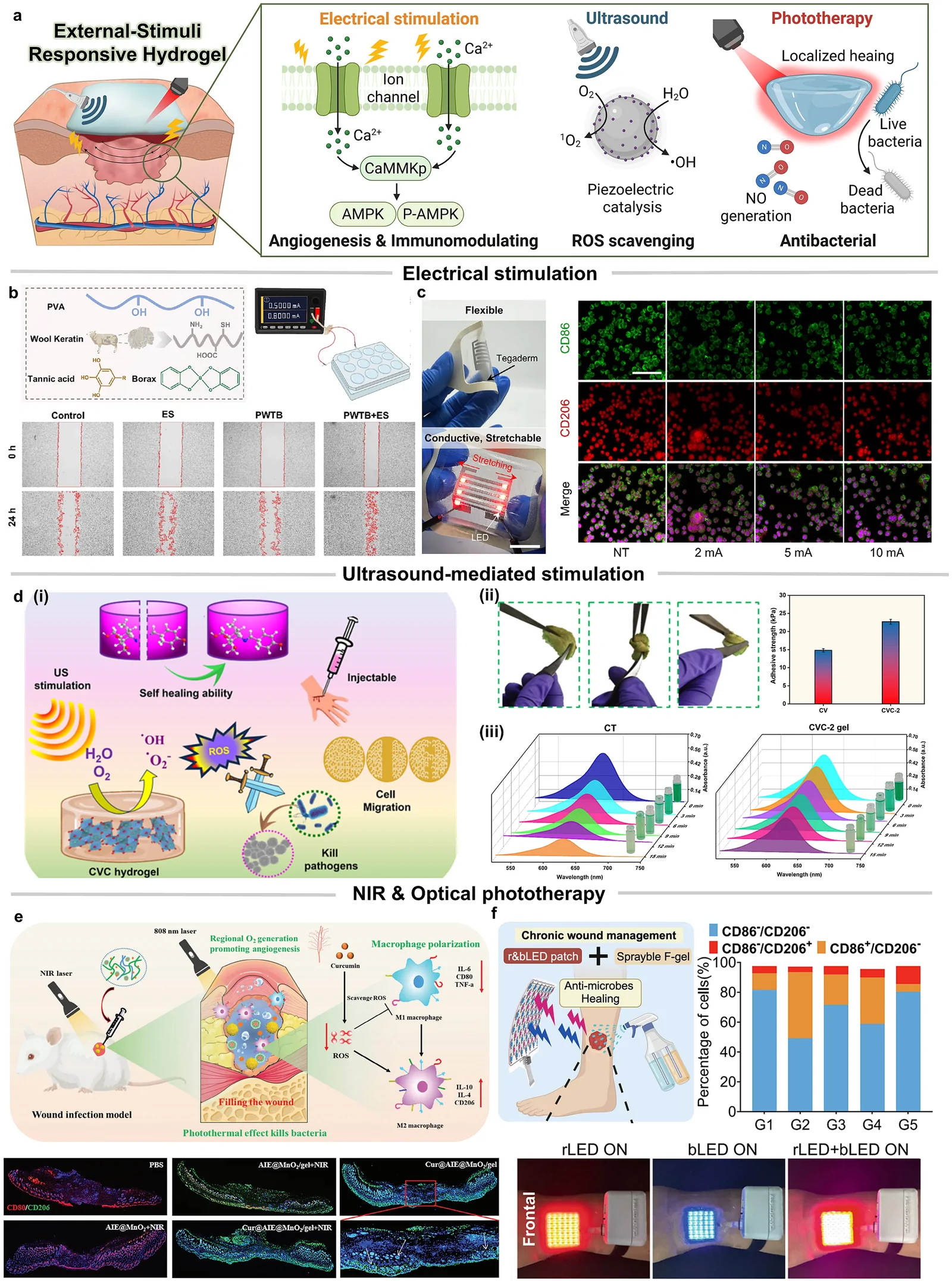
External-stimuli-responsive bioadhesive hydrogels. (**a**) Schematic of external-stimuli-responsive bioadhesive hydrogels integrating electrical stimulation, ultrasound, and phototherapy for angiogenesis, immunomodulation, and antibacterial activity. (**b**) Self-adaptive conductive hydrogel enabling electrical stimulation and real-time wound monitoring. Adapted with permission [111]. Copyright 2025, American Chemical Society. (**c**) Spray-printed LM-based electroceutical patch for optimized electrical stimulation-mediated immunomodulation in diabetic wounds. Reproduced with permission [113]. Copyright 2026 Wiley-VCH GmbH. (**d**) Injectable self-healing piezocatalytic hydrogel for ultrasound-triggered antibacterial therapy [115]. Adapted under the terms of the CC BY 4.0 License. (**e**) Thermosensitive NIR-responsive hydrogel for photothermal antibacterial and pro-regenerative effects. Adapted with permission [120]. Copyright 2024 Wiley-VCH GmbH. (**f**) Wearable dual-wavelength LED patch for antimicrobial phototherapy and enhanced wound repair [121]. Adapted under the terms of the CC BY 4.0 License

Extra-stimuli-responsive hydrogel systems are built upon a unified therapeutic paradigm in which biological effects are actively generated through the conversion of externally applied physical energy into localized biochemical signals at the wound interface [19]. As illustrated in Figure 6a, electrical fields, near-infrared (NIR) and optical phototherapy, and ultrasound waves are transduced within a bioadhesive hydrogel matrix into distinct yet complementary therapeutic cascades, including angiogenesis, immunomodulation, antibacterial activity, and redox regulation. Because the balance between anti-inflammatory and regenerative signaling shifts as the wound progresses through distinct immune microenvironment states, these external stimuli provide a means of extending inflammation resolution into the temporal domain, allowing therapeutic intensity to be actively adjusted rather than fixed by material composition alone.

This temporal control is enabled by the hydrogel–wound interface, where external energy is locally coupled to biological tissue. The hydrogel’s hydrated and mechanically compliant network facilitates efficient coupling between external energy inputs and biological tissue while spatially confining therapeutic effects. By maintaining conformal adhesion to moist and deformable wound surfaces, the hydrogel ensures stable energy transfer and localized activation. Unlike passive wound dressings that rely on diffusion-driven release of preloaded agents, external-stimuli-based hydrogel platforms enable on-demand activation with tunable intensity and temporal precision [107]. Building upon this conceptual framework, the following sections discuss in detail the material designs and mechanistic insights underlying electrical, ultrasound-mediated, and optical stimulation-triggered hydrogel therapies.

#### Electrical stimulation

Electrical stimulation (ES) has emerged as a representative external-stimuli strategy for regulating the chronic wound microenvironment, particularly in conditions marked by persistent inflammation and disrupted endogenous bioelectrical signaling [108]. During normal cutaneous injury, transepithelial potential differences generate endogenous electric fields that guide keratinocyte migration and coordinate tissue repair. In chronic wounds, however, these physiological electrical cues are weakened or dysregulated, contributing to delayed re-epithelialization and prolonged inflammatory activation. Exogenous ES aims to restore or amplify this impaired bioelectrical microenvironment in a controlled and programmable manner.

Unlike pharmacological approaches that depend on diffusion-driven delivery of biochemical agents, ES modulates cellular behavior through physical bioelectrical inputs. By altering the local electrical environment, ES influences cell migration, proliferation, angiogenic activity, and immune function, thereby reshaping the wound milieu toward a regenerative state. At the cellular level, externally applied electric fields can promote Ca^2+^ influx through membrane-associated ion channels, leading to activation of the Ca^2+^–CaMKKβ–AMPK signaling axis [109, 110]. This pathway links electrical inputs to cellular metabolism, endothelial activation, angiogenic responses, and immune regulation, including the transition of macrophages toward pro-regenerative states. ES therefore functions not merely as a directional cue for cell migration but as a bioactive physical stimulus capable of reprogramming intracellular inflammatory and regenerative signaling. In chronic inflammatory settings, such bioelectrical modulation provides a nonpharmacological means of rebalancing tissue responses without relying solely on exogenous biomolecule administration.

When integrated into soft, conformal hydrogel-based patches, ES can be delivered in a spatially confined and temporally programmable manner while maintaining intimate contact with moist and deformable wound surfaces. The hydrogel matrix ensures homogeneous electric field distribution and minimizes mechanical mismatch between the electrode and tissue, thereby improving stimulation fidelity. Such platforms allow precise tuning of current density, waveform, frequency, and directionality, parameters that critically determine cellular responses and therapeutic efficacy. Through this interface-mediated delivery of electrical energy, conductive bioadhesive hydrogels can couple stable wound contact with localized regulation of inflammation, vascularization, cell migration, and tissue regeneration. To translate this electro-immunological paradigm into a practical wound therapy platform, several groups have engineered mechanically compliant and electrically conductive hydrogel systems capable of delivering stable electric fields under physiological conditions.

In a recent study, Fang *et al*. developed a bioactive, self-adaptive conductive hydrogel platform (PWTB) composed of poly(vinyl alcohol) (PVA), wool keratin (WK), tannic acid (TA), and borax, designed to integrate ES therapy [111]. The hydrogel network was formed through dynamic borate ester bonds between PVA and borax, reinforced by catechol-borate coordination and hydrogen bonding involving TA and WK (Figure 6b). This architecture endowed the material with mechanical compliance, intrinsic ionic conductivity (~1 mS cm^−1^), self-healing capability, and conformal adaptability to irregular wound geometries. For *in vitro* evaluation, a 24-h scratch assay was conducted under direct current (DC) stimulation. L929 fibroblasts treated with PWTB hydrogel extract exhibited significantly enhanced migration compared to nonstimulated controls. Notably, 0.5 mA DC produced the most pronounced wound closure, and the combination of PWTB extract and ES further amplified cell migration relative to ES alone, indicating a synergistic pro-migratory effect.

Beyond bulk conductive hydrogels, electric field (EF) effects have also been amplified through interdigitated hydrogel-based electrode architectures. Wang *et al*. developed a flexible electric patch (ePatch) that integrates silver nanowire (AgNW)-based conductive networks with methacrylated alginate (MAA) hydrogel electrodes on a soft PU substrate [112]. In this design, AgNWs were embedded within the hydrogel matrix to establish percolated conductive pathways, enabling efficient EF transmission while preserving mechanical flexibility. The MAA hydrogel provided a hydrated and ionically conductive environment, ensuring intimate interfacial contact with moist and irregular wound surfaces. This composite architecture minimized mechanical mismatch between the electrode and tissue, thereby maintaining homogeneous EF distribution even under bending and deformation. *In vitro* scratch assays demonstrated significantly accelerated NIH 3 T3 fibroblast migration under EF stimulation compared to nonstimulated controls, with cells exhibiting pronounced anisotropic alignment indicative of cytoskeletal polarization. Beyond enhanced motility, EF stimulation upregulated the secretion of pro-regenerative growth factors, including VEGF-A and TGF-β, supporting angiogenic activation and matrix remodeling.

While these studies established the regenerative capacity of EF stimulation, parameter heterogeneity across electroceutical platforms limited mechanistic precision and translational reproducibility. Addressing this limitation, Kim *et al*. systematically optimized ES parameters using a spray-printed PVA/liquid metal (LM)–based electroceutical (SCOPE) patch [113]. The patch consisted of a spray-deposited conductive LM network embedded within a flexible PVA-based matrix and laminated onto a Tegaderm substrate, enabling intimate adhesion, mechanical flexibility, and stable current delivery under stretching and bending conditions (Figure 6c). To explore immunomodulatory effects, the authors employed an *in vitro* macrophage polarization model and screened key stimulation variables, including current intensity, duration, frequency, and directionality. Immunofluorescence staining of CD86 (M1 marker, green) and CD206 (M2 marker, red) revealed a clear intensity-dependent modulation of macrophage phenotype across stimulation conditions (NT, 2, 5, and 10 mA). In the nontreated (NT) group, macrophages exhibited strong CD86 expression, consistent with an inflammatory M1 phenotype. Upon application of 2 mA direct current stimulation (4 h/day), CD86 expression was markedly reduced while CD206 levels were preserved, indicating selective suppression of pro-inflammatory activation without excessive induction of M2 polarization. The spray-printed SCOPE platform thus exemplifies how mechanically compliant, stretchable electroceutical patches can serve as precision-controlled electro-immunological interfaces for chronic wound therapy.

Despite the rapid advancement of research on ES-integrated hydrogel systems in recent years, several translational challenges remain. Electrical dose–response relationships are highly sensitive to current density and stimulation duration, indicating a narrow therapeutic window for immunomodulation. Furthermore, stable and homogeneous current distribution requires intimate hydrogel-tissue adhesion and mechanical compliance to avoid field heterogeneity. In addition, effective stimulation fidelity depends on low interfacial impedance and proper impedance matching between the hydrogel electrode and tissue. Long-term biocompatibility, electrochemical stability, and reproducibility under dynamic wound conditions must also be carefully optimized. By restoring bioelectrical signaling, guiding immune-cell polarization, enhancing stromal-cell migration, and enabling wireless or self-powered operation, these platforms move beyond passive conductivity toward actively controlled, precision electroceutical therapies for chronic wound management.

#### Ultrasound-mediated stimulation

Ultrasound-mediated hydrogel systems represent a distinct yet parallel external-stimuli strategy, in which acoustic energy is converted into chemical and electrical cues capable of reshaping the infected chronic wound microenvironment. Unlike ES, which primarily restores bioelectrical signaling, ultrasound enables deep-tissue penetration and remote activation, offering a noninvasive modality particularly suited for infected and biofilm-laden wounds [114]. Through piezoelectric catalysis and sonodynamic mechanisms, ultrasound-responsive hydrogels can generate ROS, release therapeutic gases, and even induce mechanical contraction, thereby integrating antimicrobial, immunomodulatory, and structural wound-closure functions within a single platform.

Specifically, ultrasound-mediated hydrogel systems convert acoustic energy into therapeutic redox signals at the wound interface, thereby enabling spatially confined antibacterial and immunomodulatory regulation. Devi *et al*. engineered an injectable and self-healing piezocatalytic hydrogel (CVC) by incorporating calcium titanate (CaTiO₃, CT) nanoparticles into a dynamic chitosan/vanillin (CV) polymer network (Figure 6d(i)) [115]. The hydrogel matrix was formed via reversible Schiff-base linkages between chitosan and vanillin, reinforced by hydrogen bonding interactions, enabling strong tissue adhesion and rapid structural recovery under mechanical deformation (Figure 6d(ii)). Upon ultrasound irradiation, the embedded CT nanoparticles underwent stress-induced polarization, leading to electron–hole separation and subsequent ROS generation (•OH and O₂•^−^) through interfacial redox reactions with water and dissolved oxygen (Figure 6d(iii)). The ultrasound-dependent ROS production significantly enhanced antibacterial efficacy against both Gram-positive and Gram-negative bacteria, while maintaining high fibroblast viability and promoting cell migration.

Moreover, Roy *et al*. developed a Cu/Zn co-doped BaTiO₃ (Cu_5_Zn_5_@BTO) piezoelectric platform that converts ultrasound-induced mechanical stimulation into surface charge polarization [116]. This polarization drives electron–hole separation, enabling catalytic reactions with water and dissolved oxygen to generate ROS (•OH and •O₂^−^). The defect-engineered Cu_5_Zn_5_@BTO nanoparticles were incorporated into a thermoresponsive Pluronic F-127 hydrogel, forming a conformal and skin-adhesive matrix capable of maintaining intimate contact with moist wound surfaces. Beyond serving as a mechanical scaffold and moisture-retaining interface, the hydrogel regulates nanoparticle dispersion, Cu^2+^/Zn^2+^ ion release, and spatiotemporal ROS activation under ultrasound exposure. Electron Paramagnetic Resonance (EPR) spectroscopy using DMPO as a spin-trapping agent confirmed ultrasound-dependent radical formation, with characteristic •OH and •O₂^−^ signals observed exclusively in the Ultrasound-ON state. This acoustically triggered piezodynamic system enables localized antibacterial and anti-inflammatory activity while minimizing continuous oxidative stress, illustrating how hydrated nanocomposite hydrogels can function as externally controllable redox therapeutic interfaces for chronic wound management.

Building upon the piezodynamic ROS-generating framework, Chi *et al*. developed a multifunctional ultrasonic triple-responsive hydrogel (UTGel) that integrates ROS generation, CO release, and membrane-disruptive antibacterial mechanisms within a single ultrasound-activated platform [117]. CO-modified piezoelectric metal–organic framework nanoparticles (CO-PMOF) were incorporated into a thermoresponsive and adhesive polymer network composed of quaternary ammonium–functionalized hydrogel (QTHP) and supporting crosslinked polymer chains, forming a stretchable and conformal wound-contacting matrix. Upon ultrasound irradiation, the embedded CO-PMOF undergoes polarization-induced charge separation, driving ROS production through interfacial redox reactions. Concurrently, ultrasound-induced local heating destabilizes metal–carbonyl coordination bonds, enabling controlled CO release. Moreover, the QTHP electrostatically interacts with bacterial membranes, further enhancing structural disruption. Scanning electron microscopy (SEM) revealed that, while phosphate-buffered saline (PBS)–treated bacteria retained intact morphology, ultrasound-activated CO-PMOF induced partial membrane damage, and UTGel under ultrasound caused severe structural collapse and membrane rupture in both MRSA and *E. coli*. This synergistic ROS-CO-membrane disruption mechanism significantly amplified antibacterial efficacy compared to single-component controls.

These studies demonstrate that ultrasound-responsive adhesive hydrogels function as multifunctional energy-transducing interfaces rather than simple ROS-generating materials. By integrating piezoelectric polarization, defect-mediated redox catalysis, gas release, and membrane-active polymer networks within conformal hydrogel matrices, acoustic stimulation can be translated into spatially confined antibacterial and immunomodulatory outputs. Importantly, the adhesive and hydrated nature of the hydrogel ensures stable wound contact, efficient acoustic coupling, and localized confinement of reactive species, thereby reducing off-target oxidative damage. Such platforms highlight the potential of ultrasound-mediated adhesive hydrogels to provide remotely controllable, spatiotemporally programmable therapeutic regulation in complex chronic wound environments.

#### NIR and optical phototherapy

Phototherapy represents a noninvasive physical strategy capable of simultaneously exerting antibacterial and immunomodulatory effects in infected chronic wounds [118]. In particular, blue light–mediated photodynamic therapy and red/NIR-based photobiomodulation reprogram the wound microenvironment through distinct yet complementary mechanisms [119]. Photodynamic therapy induces ROS generation via photoactivation of sensitizers, thereby eliminating bacteria and biofilms, whereas photobiomodulation enhances mitochondrial respiratory chain activity to restore cellular energy metabolism. When integrated with adhesive hydrogel interfaces, these optical signals can be spatially localized and temporally controlled, enabling precise immunomodulatory regulation at the wound site.

For example, Wu *et al*. developed a photothermal–photodynamic composite system based on an injectable, thermosensitive chitosan hydrogel matrix encapsulating AIEgen-based photosensitizer nanoparticles (Cur@AIE@MnO₂/gel) (Figure 6e) [120]. Upon 808 nm NIR irradiation, the hydrogel generated ROS while simultaneously inducing localized photothermal heating. Notably, the hydrogel exhibited shear-thinning injectability and tissue-adhesive properties, allowing *in situ* gelation and intimate contact with the wound bed. In an infected wound model, this dual mechanism effectively suppressed bacterial growth and reshaped the immune microenvironment. Immunofluorescence staining (CD86/CD206) revealed that, compared with the PBS control, the AIE@MnO₂-gel + NIR group exhibited reduced CD86^+^ (M1) macrophage expression and increased CD206^+^ (M2) macrophage polarization. These findings indicate that NIR-activated adhesive hydrogels function not only as antibacterial agents but also as redox-mediated immunoregulatory interfaces that promote macrophage phenotype transition.

This concept was further extended by Li *et al*., who combined a sprayable fibrin gel (F-gel) with a wearable dual-wavelength LED patch [121]. The F-gel, formed via a thrombin–fibrinogen reaction, serves as an adhesive hydrogel matrix and incorporates blue light–responsive thymoquinone (TQ) and red light–synergistic Nicotinamide Adenine Dinucleotide (NADH). Under blue LED irradiation (470 nm), TQ is photoactivated to generate ROS, leading to pronounced membrane disruption of MRSA and *E. coli*, as confirmed by SEM images (Figure 6f). Furthermore, red LED irradiation (630 nm) enhances mitochondrial complex IV activity through interaction with NADH, thereby promoting ATP production and metabolic recovery in macrophages and endothelial cells. In the immune cell quantification analysis, groups G1–G5 correspond to untreated control, blue LED alone, red LED alone, dual LED treatment, and optimized combination therapy, respectively. Notably, the dual LED group demonstrated the most significant decrease in CD86^+^/CD206^−^ (M1) macrophages and a marked increase in CD86^−^/CD206^+^ (M2) macrophages, suggesting that simultaneous photodynamic therapy maximizes immune reprogramming toward a regenerative phenotype.

Importantly, the therapeutic efficacy of optical systems is significantly enhanced when integrated with adhesive hydrogel interfaces. By conforming tightly to moist and irregular wound surfaces, hydrogel matrices ensure more uniform light transmission, localized retention of photoactive components, and mechanical stabilization of fragile tissue. In this configuration, the adhesive hydrogel functions as a functional interfacial platform that converts optical energy into spatially confined biochemical signals, including ROS generation, mitochondrial activation, and immune modulation. Nevertheless, to fully realize their translational potential, further investigations are required to optimize light penetration depth, spatiotemporal regulation of ROS generation, and long-term interfacial stability in dynamic wound environments. In particular, strategies that improve light-tissue coupling efficiency, prevent off-target oxidative damage, and maintain photosensitizer stability under prolonged irradiation will be essential for advancing these systems toward clinically reliable phototherapeutic platforms.

External-stimuli-integrated bioadhesive hydrogel platforms represent a convergent therapeutic strategy in which externally applied physical energy is transduced into spatially confined biochemical outputs at the wound interface. Although electrical, ultrasound-mediated, and optical stimuli differ in activation modality, they share a common mechanistic principle: energy conversion within a conformal, hydrated hydrogel matrix that enables localized immune modulation, antibacterial activity, angiogenic stimulation, and redox regulation. To comprehensively compare these systems, Table 3 summarizes their material composition, specific external stimuli conditions, wet adhesion strength, immunomodulatory outcomes, targeted wound healing models, and translational potential. However, it is critical to contextualize these outcomes within their specific experimental models rather than making direct comparisons of generalized metrics like wound closure percentages. For instance, ES platforms (e.g. PWTB, ePatch) are primarily evaluated in uninfected models to demonstrate accelerated tissue regeneration and angiogenic marker upregulation. In contrast, ultrasound and phototherapy-based systems (e.g. Cu5Zn5@BTO, F-gel, and LED patch) are typically tested in severe, highly infected (e.g. MRSA) burn or diabetic wound models. In these infected models, the primary immunological focus is the suppression of infection-driven M1 macrophage polarization (decreased CD86) and the subsequent transition to a reparative M2 state (increased CD206). Consequently, the therapeutic efficacy and biological outcomes of these systems must be evaluated based on their target wound etiology.

**Table 3:** External-stimuli-responsive bioadhesive hydrogel for wound healing

Material (composition & cey feature)	External-stimuli (type & condition)	Wet adhesion strength	Immunomodulatory outcome	Wound healing model & Key result	Translational potential	Refs
PWTB hydrogel Borate-crosslinked PVA/wool keratin/tannic acid (TA).	Electrical stimulation DC-CC, 0.5 mA, 15 min.	~8 kPa (porcine skin)	• Local/systemic IL-6 & TNF-α↓	• Mouse surgical model (10 mm) • 99.96% wound closure at Day 14 (vs control 82.0%) • Enhanced COL-1 deposition	• Resistance-based sensing validated in postoperative human clinical trials • Trailing signal tracks abscess (<50% resistance) *vs* healing (>200%)	[111]
AgNW/MAA ePatch AgNW network in methacrylated alginate (MAA) on PU substrate.	Electrical stimulation Pulsed EF; continuous 6 V, 10 Hz, 1 ms pulse width.	NR	• VEGF-A & TGF-β upregulation • Inflammatory cell infiltration phase shortened • T-cell (CD3)↓ 79.5%, B-cell (CD79a)↓ 55.4% at Day 7	• Rat full-thickness model (8 mm) • Open wound area at Day 4: 25.7% (vs control 57.9%) • Closed within 7 days (vs normal 20 days)	• Portable wireless EF generator (15 g) • AgNW intrinsic antibacterial block without toxicity • Rodent validation only	[112]
SCOPE patch Spray-printed liquid metal (LM) network via Ga₂O₃ passivation in PVA matrix on Tegaderm.	Electrical stimulation Unidirectional AC with offset (Uni-AC); 2 mA, 400 Hz, 4 h/day.	NR	• CD86 & iNOS selective suppression • TNF-α↓; IL-10↑	• Type 1 diabetic mouse wound model (6 mm) • Open area remaining at Day 3: 23.4% (DC)/34.7% (AC) *vs* NT 66.8% • CD31+ neovascularization enhanced by Day 12	• Highly stretchable network (stable over 1000 cycles) • Current platform is wired; requires wireless adaptation for clinical scaling	[113]
CVC-2 hydrogel Calcium titanate (CaTiO₃, CT) nanoflakes (2 wt%) in chitosan/vanillin matrix.	Ultrasound-mediated Ultrasonic irradiation/sonodynamic therapy (SDT); 40 kHz, 10 min.	23 kPa	• Controlled piezocatalytic ROS generation (•OH, O₂•−) • Vanillin matrix provides antioxidant protection to eliminate collateral healthy cell damage	• Reached ~80% scratch closure within 24 h	• Autonomous self-healing (15 min) via Schiff base bonds; injectable network • Completely lacks in vivo animal wound validation data	[115]
Cu_5_Zn_5_@BTO gel Cu/Zn co-doped BaTiO₃ (BTO) nanocrystals in Pluronic F-127.	Ultrasound & body movement Dual piezodynamic-chemodynamic activation; US (15 kHz) or body movements (1–6 Hz).	NR	• M1-to-M2 macrophage shift (iNOS↓, Arg1↑) • Flow cytometry: CD86↓ ~30%, CD206↑ ~20% • Suppresses pro-inflammatory IL-6, IL-1β, TNF-α	• In vivo MRSA-infected burn mouse model • Accelerated wound healing & epidermal regeneration by Day 10 • Fenton-like CDT reaction extends radical generation	• Generates electrical voltage impulses under low-frequency natural body movements (1–6 Hz) • High blood safety: no large-animal data	[116]
UTGel CO-modified carnosine-zinc PMOF in crosslinked QTHP/HA-SH.	Ultrasound-Mediated Acoustic excitation: 1.5 W/cm^2^, 1.0 MHz, 50% duty cycle, 10 min. Triggers simultaneous ROS, CO release, and contraction.	5.5 kPa (Porcine skin)	• Mitigates Dysregulated inflammation • Day 7: local pro-inflammatory IL-1β (0.55) & TNF-α (0.06) drastically suppressed • Day 18: α-SMA↑ 5.1-fold (myofibroblast polarization)	• MRSA-infected Full-thickness diabetic mice (10 mm) • US triggers 52.4% centripetal gel shrinkage within 4 min • 96% ± 3% wound closure at Day 12; 100% complete closure by Day 18	• Reutilizes Ultrasound waste heat into active mechanical wound self-closure • Hemostatic capability: cuts blood loss by ~75%; arrests bleeding in 113–118 s	[117]
Cur@AIE@MnO₂/gel Curcumin/AIEgen/MnO₂ encapsulated in thermosensitive chitosan network.	NIR laser irradiation 808 nm NIR laser (1.5 W/cm^2^, 10 min); triggers localized PTT hyperthermia (57.5°C *in vivo*) & ROS.	55 kPa (porcine skin)	• M1-to-M2 shift (CD80/TNF-α/IL-6↓; CD206/IL-10↑) • Upregulates anti-inflammatory genes Tnip3 & Traf5 • Downregulates pro-inflammatory markers (Tnfrsf12a, Il15)	• Excisional wound model infected with *S. aureus* in mice • Near-complete wound closure and hair follicle regeneration by Day 10	• Injectable thermosensitive adaptability to irregular geometries • MnO₂ nanosheets continuously Catalyze tissue H₂O₂ into oxygen to relieve hypoxia • Heavy reliance on external NIR laser infrastructure	[120]
F-gel + LED patch Sprayable fibrin gel co-loaded with TQ and NADH, paired with a stretchable wearable LED patch.	Dual phototherapy Blue LED (470 nm, 50 mW/cm^2^, 12 min) for PDT + Red LED (630 nm, 10 mW/cm^2^, 5 min) for PBM.	NR	• Inhibits NF-κB p65 nuclear translocation & iNOS expression • Promotes M2 phenotype (CD206↑) and suppresses M1 phenotype (CD86↓) • NADH restores mitochondrial OXPHOS	• Infected diabetic wounds in mice and Panama minipigs (1.5 cm) • Completely eradicated acute MRSA/Pa infection • Minipig models achieved accelerated scab detachment & neovascularization by Day 20	• Mobile-app controlled wearable device validated in large animals • Safe home care execution: built-in temperature alarms with automatic power-off at 42°C	[121]

*CCT* calcium titanate, *LM* liquid metal, *PVA* poly(vinyl alcohol), *AgNW* silver nanowire, *MAA* methacrylated alginate, *PU* polyurethane, *TA* tannic acid, *SDT* sonodynamic therapy, *AC* alternating current, *EF* electric field, *DC* direct current, *ROS* reactive oxygen species, *iNOS* inducible nitric oxide synthase, *TNF-α* tumor necrosis factor alpha, *IL* interleukin, *VEGF* vascular endothelial growth factor, *TGF-β* transforming growth factor beta, *NT* no treatment, *COL-1* collagen type I, *LED* light-emitting diode, *TQ* thymoquinone, *AIE* aggregation-induced emission, *NIR* near-infrared, *PTT* photothermal therapy, *PMOF* porphyrinic metal–organic framework, *HA-SH* thiolated hyaluronic acid, BTO, barium titanate, *PDT* photodynamic therapy, *PBM* photobiomodulation, *US* ultrasound, *OXPHOS* oxidative phosphorylation, *α-SMA* alpha-smooth muscle actin, *MRSA* methicillin-resistant *Staphylococcus aureus*, *CDT* chemodynamic therapy

### Sensing-integrated bioadhesive hydrogels

Conventional wound dressings and patch-type therapeutic systems are typically applied for extended periods, yet accurately evaluating the degree of tissue recovery during treatment remains challenging [122]. Premature removal of a dressing before sufficient healing has occurred may cause unintended tissue damage, disrupt newly formed granulation tissue, and impose significant pain and discomfort on patients. Conversely, prolonged application without proper assessment may delay necessary intervention. Accordingly, there has been a growing demand for platforms capable of real-time, quantitative evaluation of wound status directly at the tissue interface. Because effective immune microenvironment reprogramming depends on aligning therapeutic activity with the wound’s actual inflammatory status, such real-time information can further guide the structural, bioactive, and stimuli-responsive strategies described above, closing the loop between diagnosis and immunomodulatory intervention. Moving beyond hydrogel systems focused solely on therapeutic functions, recent advances have incorporated diagnostic capabilities that enable continuous monitoring of the wound microenvironment [123, 124, 125].

**Figure 7:**
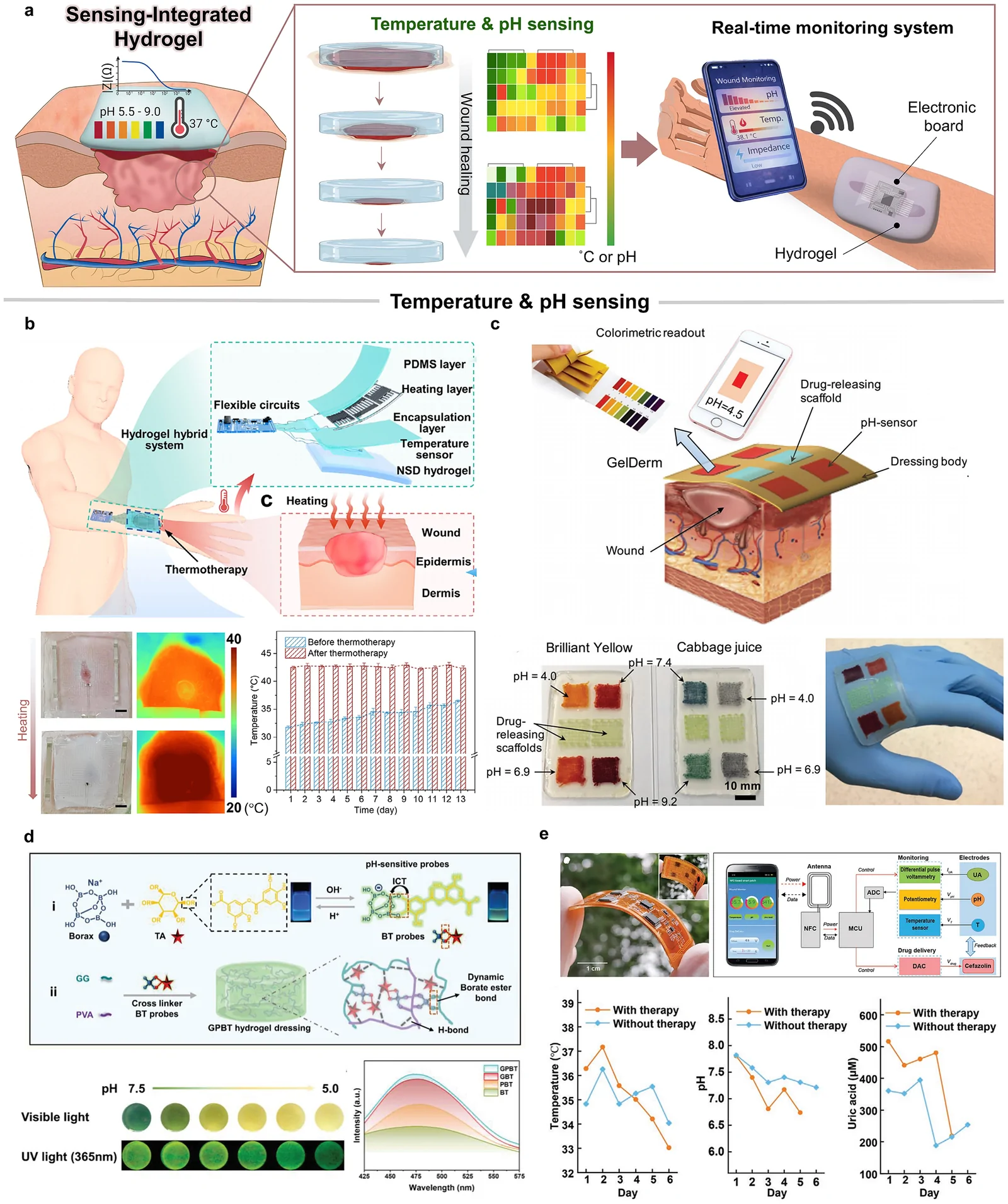
Sensing integrated bioadhesive hydrogels. (**a**) Schematics of a sensing-integrated hydrogel enabling continuous wound temperature and pH tracking with wireless transmission for remote monitoring. (**b**) Wireless thermoresponsive hydrogel patch with customizable adhesion and closed-loop temperature control for conformal thermotherapy [127]. Adapted under the terms of the CC BY 4.0 License. (**c**) Colorimetric pH-responsive hydrogel dressing incorporating a drug-releasing scaffold and pH-sensing layer within a conformal GelDerm configuration for wound monitoring and stage-matched drug delivery. Reproduced with permission [129]. Copyright 2017 Wiley-VCH GmbH. (**d**) Boron-based probe-crosslinked GG/PVA theranostic hydrogel (GPBT) providing colorimetric pH visualization and stage-matched chronic wound modulation. Reproduced with permission [130]. Copyright 2023, Wiley-VCH GmbH. (**e**) Battery-free, NFC-enabled smart wound dressing integrating temperature and pH sensing with electrically controlled antibiotic delivery [131]. Adapted under the terms of the CC BY 4.0 License

Chronic wounds exhibit pronounced spatial and temporal heterogeneity, with fluctuations in pH, temperature, and electrical conductivity across inflammatory and regenerative phases. Sensing-integrated hydrogel systems address this complexity by continuously detecting biochemical and biophysical changes within the wound bed and converting them into quantifiable electrical, optical, or colorimetric signals. By embedding sensing components within soft, adhesive matrices, these systems maintain conformal contact with moist and irregular wound surfaces, thereby functioning as bioelectronic interfaces that link tissue conditions to digital readouts and potential therapeutic feedback. Parameters such as pH, temperature, and impedance can be wirelessly transmitted to external devices for real-time display, effectively transforming hydrogel dressings into wearable monitoring systems (Figure 7a) [126–131].

#### Temperature and pH sensing–integrated hydrogels

Temperature serves as a representative biophysical marker that sensitively reflects inflammatory burden, infection status, and barrier restoration during wound healing [126]. Local temperature elevation is closely associated with bacterial colonization and inflammatory activation, whereas tissue impedance quantitatively reflects epithelial continuity, hydration level, ionic conductivity, and structural recovery. Integrating these sensing modalities within adhesive hydrogel interfaces enables continuous, noninvasive, and mechanically stable monitoring of the evolving wound microenvironment.

Yang *et al*. reported a wireless hydrogel-based thermotherapy platform integrating flexible electronics with an ionically conductive hydrogel interface [127]. The multilayer system consists of PDMS encapsulation, flexible circuitry with a resistive heater and temperature sensor, and a nonswelling dual-network (NSD) hydrogel as the skin-contacting layer (Figure 7b). The NSD hydrogel, composed of covalently crosslinked polyacrylamide (PAM) and ionically crosslinked alginate, provides mechanical toughness, compliance, and strong wet adhesion while maintaining structural stability without excessive swelling. Temperature is monitored via a resistive sensor, enabling real-time feedback-controlled heating. The conformal hydrogel interface ensures efficient heat transfer and accurate temperature readout. Infrared imaging confirmed uniform temperature distribution and controlled elevation from physiological (~30°C–32°C) to therapeutic ranges (~40°C–42°C), with stable and reproducible performance over repeated cycles for up to 13 days. These results underscore the importance of the adhesive, mechanically compliant hydrogel in maintaining thermal homogeneity and long-term signal stability.

In parallel with temperature sensing, pH is a clinically significant biomarker in chronic wound management, as infected wounds typically shift toward alkaline conditions (pH > 7.5), whereas healing tissues gradually return to neutral or slightly acidic environments [128]. Adhesive hydrogel-based pH-sensing systems, therefore, provide a powerful strategy for early infection detection and spatial mapping of pathological regions directly at the wound interface. For instance, Mirani *et al*. reported a colorimetric pH-responsive hydrogel dressing designed as a multifunctional wound management platform [129]. The system consisted of a multilayer structure including a drug-releasing scaffold, a pH-sensing layer, and a dressing body integrated within a conformal GelDerm configuration (Figure 7c). The hydrogel was engineered to adhere to moist wound surfaces while exhibiting distinct color changes across a wide pH range. Clear chromatic differentiation was observed at pH 4.0, 6.9, 7.4, and 9.2 using indicator systems such as Brilliant Yellow and cabbage juice extracts, demonstrating sensitivity across physiologically relevant conditions. The colorimetric response could be quantitatively analyzed through smartphone-based RGB extraction, enabling conversion of visual signals into numerical pH values. Importantly, the adhesive hydrogel interface maintained stable contact with irregular and exudative wound surfaces, minimizing signal distortion and enabling reliable *in situ* sensing. By integrating pH detection with drug-release functionality, this platform illustrated the potential for responsive wound management guided by biochemical status.

Building upon colorimetric pH dressings, Zhang *et al*. developed a boron-based theranostic hydrogel (GPBT) that integrates visual pH monitoring with stage-matched wound therapy [130]. Unlike physically mixed dye systems, the boron-based probe formed dynamic borate ester bonds within the hydrogel network, enabling stable, uniform, and leakage-free pH responsiveness (Figure 7d). The dressing exhibited distinct color transitions corresponding to alkaline-infected stages and acidic healing phases, which could be quantitatively analyzed via smartphone-based RGB extraction. The developed hydrogel was engineered to match the dynamic healing process of chronic wounds: under early alkaline conditions, controlled tannic acid release promoted M2 macrophage polarization and angiogenesis, while progressive acidic shifts triggered programmed hydrogel disassembly to support tissue regeneration.

Furthermore, Xu *et al*. developed a battery-free, wireless smart wound dressing capable of simultaneous temperature and pH sensing, enabling closed-loop wound management [131]. The system consists of a reusable NFC-enabled flexible circuit layer and a disposable stretchable electrode array fabricated on a polyimide (PI) substrate with serpentine copper interconnects and PDMS encapsulation. Temperature was measured using an integrated semiconductor temperature sensor, while pH was monitored potentiometrically via a PANI-modified carbon working electrode with an Ag/AgCl reference stabilized by a PVB/NaCl coating (Figure 7e). The hydrogel-contacted interface ensured conformal attachment to the wound surface, improving thermal transfer and electrochemical signal stability. *In vivo* studies further highlighted the importance of multiplexed sensing. Infected wounds showed elevated temperature and alkaline pH, whereas wounds treated with electrically controlled cefazolin release (loaded in a PPy-based drug delivery electrode) exhibited a more rapid decline in temperature and a faster shift toward near-neutral pH. These coordinated thermal and biochemical changes confirm that integrated dual-parameter monitoring enhances infection assessment accuracy and supports feedback-guided therapeutic intervention.

While temperature and pH sensing primarily reflect inflammatory burden and infection status, impedance serves as a complementary biophysical biomarker that quantitatively evaluates tissue barrier restoration and structural regeneration. Shin *et al*. reported a fully hydrogel-based electronic skin (e-skin) platform capable of impedance-based wound monitoring alongside ES [132]. The all-hydrogel architecture comprising a PHEA substrate, Ag-flake conductive hydrogel interconnects, PEDOT: PSS electrodes, and a PDA-based adhesive hydrogel layer provides tissue-like softness and low interfacial impedance, enabling stable and high-fidelity bioelectrical measurements. Frequency-dependent impedance analysis revealed lower impedance at wounded sites compared to intact skin, with progressive recovery observed during healing. Notably, wounds treated with ES exhibited a more rapid impedance increase, confirming accelerated restoration of structural integrity. In addition, Jiang *et al*. reported a wireless, closed-loop smart bandage system that combines sensors and stimulators within a flexible bioelectronic platform [133]. This system incorporates temperature and impedance sensors with drug-delivery or electrical-stimulation modules, enabling feedback-controlled modulation of therapeutic intensity based on real-time physiological signals. Wireless data transmission allows continuous tracking of wound status, while *in vivo* studies demonstrated accelerated wound closure and reduced inflammatory responses.

Despite these advances, the long-term analytical reliability of sensing-integrated wound dressings remains insufficiently characterized. As summarized in Table 4, most studies report response time, mechanical cycling durability, storage stability, or short-term performance in biological fluids, but these measurements do not necessarily predict sensor behavior during continuous exposure to protein-rich and enzyme-containing wound exudate. Quantitative seven-day assessments of baseline drift, sensitivity retention, hysteresis, and biofouling-induced signal degradation are rarely reported; among the reviewed electrochemical systems, a pH drift of 0.41 mV h^−1^ was reported, but prolonged exudate exposure was not systematically evaluated. Moreover, although several platforms were tested in animal wound models, most studies demonstrated correlations between sensor outputs and healing or infection status rather than formal *in vivo* calibration against reference measurements. Future studies should therefore evaluate sensors during continuous exposure to simulated or clinically collected wound exudate and perform *in vivo* calibration against established methods, such as microelectrodes, thermocouples, infrared imaging, or standardized biochemical assays. Collectively, these studies demonstrate that adhesive hydrogel-integrated temperature and impedance sensing platforms are advancing beyond passive monitoring systems toward robust bioelectronic interfaces capable of continuous wound assessment, quantitative structural regeneration tracking, and feedback-controlled therapeutic regulation.

**Table 4:** Comparative assessment of representative sensing-integrated bioadhesive hydrogel and smart wound-dressing platforms

Material	Sensing modality	Sensing mechanism	Operational stability duration	Performance under exudate/biofouling	*In vivo* calibration	Key limitation	Refs.
Polyacrylamide(PAM)–quaternized chitosan(QCS)–carbon quantum dots(CQD)s–phenol red hydrogel	pH (dual colorimetric: visible + UV fluorescence)	Optical color/fluorescence change read via smartphone RGB → pH equation	Reversible over 10 cycles (pH 5–8)	Reduced swelling (50% of PAM), good adhesion (3.12 kPa), and no quantitative biofouling data	Mouse wound model, matched pH test paper	Single-parameter (pH only); no long-term/biofouling stability data	[123]
α-Si nanomembrane array + drug-loaded gelatin hydrogel + IR-LEDs	Temperature (64-ch array)	Resistive, Arrhenius-law TCR (R↓ as T↑)	Stable in biofluid 15 d; stable after 1 mo & 1000 bending cycles	Fails at 96°C accelerated test (0% yield after 2 d) due to biofluid permeation through PI	Infected rat wound model, validated *vs* IR camera/thermocouple	Single-parameter (temp only); heat from the IR-LED can confound the inflammation signal	[124]
N-isopropylacrylamide(NIPAM)/SBMA/PAA-dopamine hydrogel + Ag-paste heating layer on PDMS	Temperature (therapy device, not diagnostic sensor)	Resistive heating + thermistor feedback; thermoresponsive adhesion switching	±0.1°C control; <7% resistance change after 3500 bends	N/A (therapeutic device, not exudate sensor)	Rat wound model, WHR 99.58% at Day 14	No biosensing (pH/infection); heating-only, no diagnostics	[127]
Alginate hydrogel + pH dye beads + gentamicin	pH (colorimetric)	Dye color change → RGB → pH	Response <5 min; ~2 h swelling eq.	Untested (bacterial culture only)	Not reported (*ex vivo* pig skin only)	Single param; needs smartphone	[129]
GG/PVA + boron-tannic acid probe hydrogel	pH (color+ fluore-scence)	Borate ester bond breaks w/pH → color shift	Antibacterial/antioxidant, fouling untested	Antibacterial/antioxidant, fouling untested	Rat diabetic wound	Single param; lighting-sensitive	[130]
PI electrode + rGO/AuNPs + PPy/cefazolin, NFC circuit	pH + Temp + uric acid (electrochemical) + drug delivery	Electrode potential/DPV shifts, PPy redox release	Battery-free NFC; pH drift 0.41 mV/h	Interferent-tested only, fouling untested	Rat infection model	Rigid substrate; no defined release threshold	[131]
5-layer hydrogel stack (PHEA/Ag/PEDOT:PSS/PDA/PVA)	Impedance only + EF stim/iontophoresis	Wound = ion leakage → impedance↓, 2D mapping	Tissue-like modulus, directional adhesion	Not reported	Mouse full-thickness wound	Only 1 sensor type; wired power	[132]
Battery-free NFC circuit + PEDOT:PSS/NIPAM thermoresponsive hydrogel	Impedance + Temp + closed-loop electrical stim	AC impedance, thermistor, LCST-based adhesion switch	15 cm wireless; 10 000-cycle durable; detach at 40°C	Explicitly flagged as unresolved by authors.	Mouse excisional/burn/diabetic/infection models, scRNA-seq mechanism	Only 2 sensors; scalability/cost unresolved	[133]

*PAA* poly(acrylic acid), *NIPAM* N-isopropylacrylamide, *SBMA* sulfobetaine methacrylate, *Ag* silver, *PDMS* polydimethylsiloxane, α-Si amorphous silicon, *IR* infrared, *LED* light-emitting diode, *PAM* polyacrylamide, *QCS* quaternized chitosan, *CQDs* carbon quantum dots, *UV* ultraviolet, *RGB* red–green–blue, *TCR* temperature coefficient of resistance, *PI* polyimide, *WHR* wound healing rate, *NFC* near-field communication, *PDA* polydopamine; *PVA* poly(vinyl alcohol), *GG* guar gum, *EF* electric field, *AC* alternating current, *LCST* lower critical solution temperature, *DPV* differential pulse voltammetry

### Future perspectives and challenges

**Figure 8:**
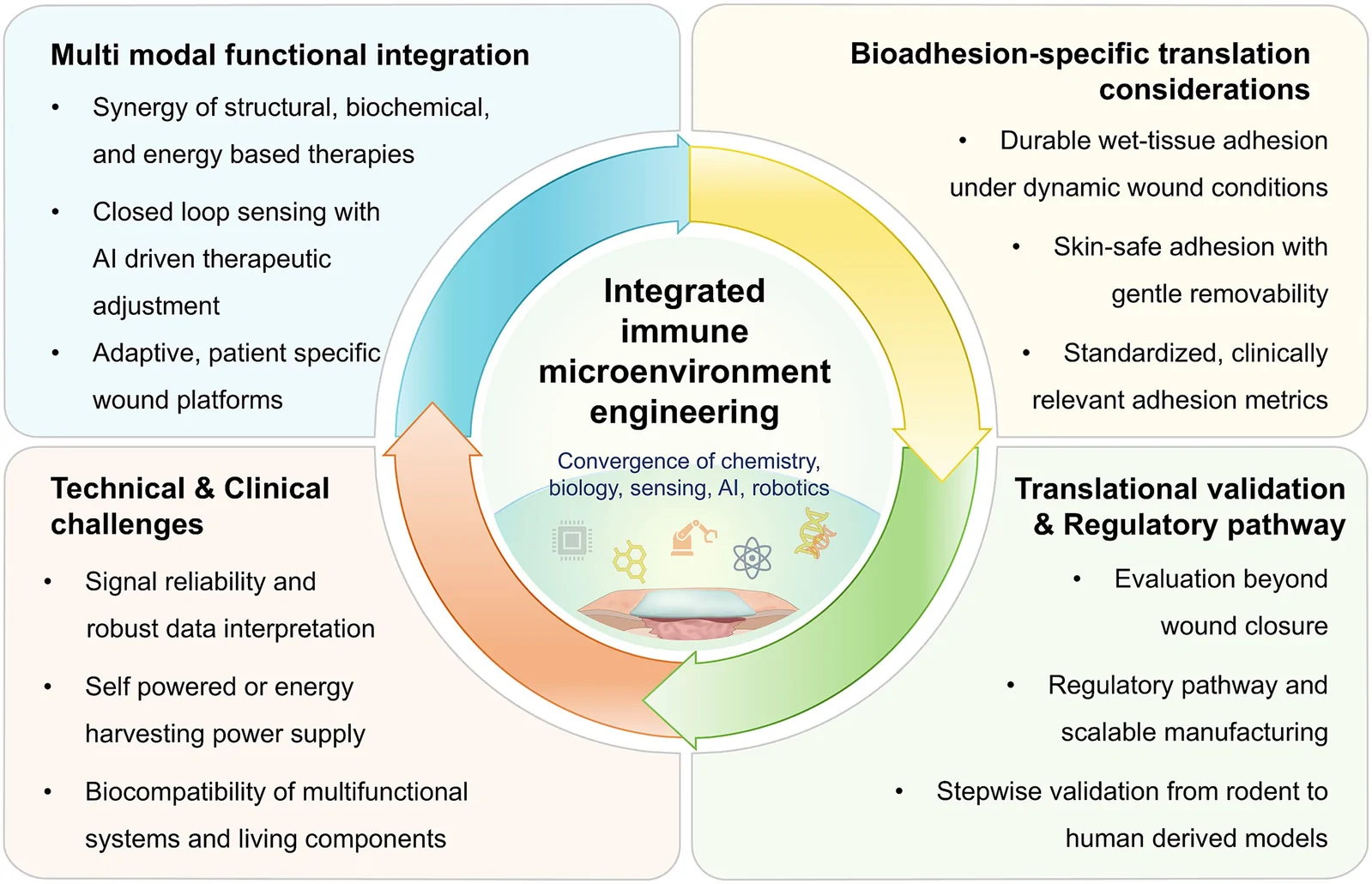
Future perspectives and challenges. Integrated immune microenvironment engineering for advanced wound care: multi-modal functional integration, bioadhesion translation considerations, technical/clinical challenges, and translational validation and regulatory pathway

The evolution of bioadhesive hydrogels has entered a transformative phase in which material science, immunology, and digital technology no longer operate in parallel but converge to redefine wound care as a paradigm of Integrated Immune Microenvironment Engineering (Figure 8). Central to this convergence is the recognition that effective wound healing requires not merely the delivery of therapeutic agents but the systematic reprogramming of dysregulated immune signaling networks. This reprogramming spans a range of pathways, from NF-κB-driven pro-inflammatory cascades to STAT6/STAT3-mediated reparative pathways. Rather than serving as passive dressings, next-generation bioadhesive hydrogels are emerging as multifunctional platforms capable of orchestrating immune regulation, mechanical stabilization, biochemical sensing, and programmable therapeutic intervention within a single interface. To translate these conceptual advances from laboratory innovation to clinical standard of care, several critical dimensions must now be systematically addressed:

(i) The integration of multi-modal functionalities remains the primary frontier. Future platforms must go beyond simple drug delivery; they must achieve a synergy between structural integrity, biochemical signaling, energy-based therapies, and real-time sensing. The goal is to develop adaptive, patient-specific wound platforms that can dynamically respond to the fluctuating immune status of an individual’s wound. This necessitates a “closed-loop” system where sensors detect specific biomarkers of inflammation or infection, and artificial intelligence (AI)-driven algorithms immediately adjust the therapeutic output, such as modulating ES or releasing specific immunomodulators targeting defined signaling axes such as NF-κB suppression or STAT6 activation.

(ii) From a translational perspective, bioadhesion-specific considerations must be standardized. While high adhesion strength is often reported, the durability of this adhesion under the extremely dynamic and wet conditions of clinically relevant wounds is still insufficient. Moreover, a critical “adhesion paradox” must be solved: the material must provide robust, leak-proof sealing while ensuring skin-safe, gentle removability to prevent secondary trauma during dressing changes. Establishing standardized and clinically relevant metrics for evaluating adhesion in diverse physiological environments, ranging from high-pressure hemorrhages to chronic exudative ulcers, is essential for regulatory approval and clinical trust.

(iii) Technical and clinical barriers regarding the long-term reliability of multifunctional systems must be overcome. The biocompatibility of complex electronic components and living biological fillers, such as algae or stem cells, needs rigorous long-term validation. Furthermore, the power supply for active bioelectronic patches remains a challenge. Future research should prioritize self-powered systems or high-efficiency energy harvesting from the body’s own movements or thermal gradients. Ensuring signal reliability and developing robust data interpretation frameworks will be vital for making these devices practical for outpatient use.

(iv) A major limitation of the current evidence base is its predominant reliance on acute rodent wound models. Although these models are valuable for initial mechanistic screening, rodent wounds often heal rapidly through contraction and do not fully reproduce the persistent inflammation, impaired perfusion, neuropathy, biofilm-associated infection, prolonged exudation, recurrent mechanical loading, and multiple comorbidities characteristic of human chronic wounds [134]. Consequently, short-term wound-closure measurements alone should not be regarded as sufficient evidence of clinical translatability. Future validation should follow a stepwise and indication-specific framework. Initial studies should incorporate disease-relevant rodent models reflecting diabetes, aging, ischemia, pressure loading, venous dysfunction, or chronic biofilm-associated infection according to the intended wound subtype. Splinted excisional models may further reduce contraction-related bias and improve the assessment of re-epithelialization [135]. Promising platforms should subsequently be evaluated in large-animal models, particularly porcine wound models, which more closely approximate human skin architecture, thickness, mechanical behavior, and clinically relevant dressing dimensions [136]. These studies should be complemented by human-relevant platforms, including *ex vivo* human skin and vascular-, immune-, or microbiome-integrated three-dimensional wound models [137]. Across these platforms, evaluation should extend beyond gross wound closure to include re-epithelialization, tissue perfusion, immune-cell phenotypes, microbial burden, extracellular-matrix remodeling, restoration of skin appendages, recurrence, atraumatic dressing removal, and long-term local and systemic safety. No individual model is sufficient to predict clinical performance; rather, translation will require convergent evidence from disease-specific small-animal models, large-animal studies, and human-derived validation platforms.

(v) Clinical translation further requires a realistic bridge between currently available wound-care products and the multifunctional hydrogel platforms described in this review. Representative marketed hydrogel products, such as INTRASITE Gel and DuoDERM Hydroactive Sterile Gel, primarily support moisture balance, autolytic debridement, and the hydration or filling of dry and cavity wounds. These products demonstrate the clinical feasibility of relatively simple hydrogel formulations but generally do not provide active immunomodulation, integrated biochemical sensing, programmable drug delivery, or closed-loop therapy. Thus, the translational gap between established hydrogel dressings and next-generation multifunctional systems lies not only in therapeutic performance but also in fabrication complexity, regulatory classification, cost, and practical integration into clinical workflows.

Hydrogels based on established and relatively inexpensive polymers, including PVA, carboxymethylcellulose, alginate, hyaluronic acid, and chitosan, may offer clearer translational pathways because these materials have extensive clinical and commercial precedent in wound-care products. In the USA, hydrogel wound dressings without an added drug or biologic may fall within the Class I, 510(k)-exempt category under 21 CFR §878.4022, reflecting the extensive regulatory precedent already established for simple hydrogel formulations in wound care [138, 139]. However, this comparatively straightforward pathway may not apply when drugs, biologics, living cells, animal-derived materials, or active electronic modules are incorporated. Under the U.S. Food and Drug Administration (FDA) ’s 2023 proposed rule for antimicrobial wound dressings, products containing antimicrobials with low or medium antimicrobial-resistance concern were proposed to be classified as Class II devices subject to special controls and premarket notification, whereas products containing medically important antimicrobials were proposed to be classified as Class III devices requiring premarket approval [140]. Such distinctions can substantially increase the extent of safety testing, development cost, and regulatory timelines.

By contrast, emerging functional components, including liquid metals, piezoelectric metal–organic frameworks, aggregation-induced-emission luminogens, living biological materials, and integrated electronic modules, provide distinctive sensing or stimuli-responsive capabilities but currently lack well-established regulatory precedent for wound-contact applications. Their raw-material cost, batch-to-batch consistency, sterilization tolerance, degradation behavior, storage stability, and long-term biological safety also remain insufficiently standardized. The transition from hand-crafted laboratory prototypes to mass-produced, standardized medical devices, therefore, requires not only advanced fabrication approaches, such as high-throughput printing, scalable molding, 3D bioprinting, or roll-to-roll processing [141] but also simplified formulations, reproducible raw-material sourcing, validated sterilization procedures, shelf-life and packaging assessments, and regulatory-grade quality control.

Commercialization will additionally depend on manufacturing cost, reimbursement, ease of use within existing clinical workflows, and whether the added immunomodulatory, sensing, or therapeutic functions provide sufficient clinical benefit over simpler commercial dressings to justify their increased complexity and cost. Accordingly, near-term translation may favor immunomodulatory functions achievable using established polymer systems and relatively simple manufacturing processes, whereas platforms incorporating exotic functional materials, living components, or complex electronics are likely to require longer-term regulatory and manufacturing development. Based on these considerations, several research directions appear particularly promising for future development. First, stage-specific bioadhesive hydrogels should be designed to provide temporally matched immunomodulatory cues according to the progression from inflammation to regeneration and remodeling. Second, closed-loop platforms integrating real-time sensing of wound-relevant biomarkers with programmable drug delivery, electrical stimulation, or other therapeutic outputs should be further developed to enable adaptive regulation of the wound microenvironment. Third, greater emphasis should be placed on simplified, scalable, and disease-specific platforms that combine durable wet adhesion with a limited number of mechanistically validated therapeutic functions and can be evaluated using clinically relevant wound models. These directions may provide a practical roadmap for advancing bioadhesive hydrogels from multifunctional laboratory prototypes toward clinically actionable wound-care systems.

As AI and robotics become increasingly integrated into manufacturing, monitoring, and therapeutic control, smart closed-loop immune-modulating patches may ultimately become part of routine wound care. However, their adoption will require not only technological performance but also disease-relevant validation, clear regulatory pathways, cost-effectiveness, and clinically demonstrated superiority over established wound-care products.

## Conclusions

Bioadhesive hydrogels have advanced chronic wound care by establishing stable wound–material interfaces that integrate tissue adhesion with immunomodulatory, regenerative, stimuli-responsive, and sensing functions. Despite substantial progress, clinical translation remains limited by challenges including unstable adhesion under wet and dynamic conditions; uncontrolled swelling and degradation; insufficient mechanistic validation; inconsistent evaluation standards; and concerns regarding sterilization, reproducibility, manufacturing complexity, cost, and regulatory pathways. Future research should therefore focus on clinically defined and disease-specific designs tailored to wound etiology, exudate level, infection status, anatomical loading, and healing stage, while adopting standardized testing under physiologically relevant conditions and establishing causal links between material properties and immune responses. Near-term translation will likely favor simplified and scalable platforms that combine reliable wet adhesion with a limited number of well-validated therapeutic functions. In contrast, longer-term development may enable closed-loop systems that integrate continuous sensing with stage-appropriate drug delivery, electrical stimulation, phototherapy, or other controllable interventions. Ultimately, successful bioadhesive hydrogel dressings will require a balance between functional sophistication, mechanistic reliability, manufacturability, atraumatic use, cost-effectiveness, and demonstrable clinical benefit.
